# Causal relationship of hepatic fat with liver damage and insulin resistance in nonalcoholic fatty liver

**DOI:** 10.1111/joim.12719

**Published:** 2017-12-27

**Authors:** P. Dongiovanni, S. Stender, A. Pietrelli, R. M. Mancina, A. Cespiati, S. Petta, S. Pelusi, P. Pingitore, S. Badiali, M. Maggioni, V. Mannisto, S. Grimaudo, R. M. Pipitone, J. Pihlajamaki, A. Craxi, M. Taube, L. M.S. Carlsson, S. Fargion, S. Romeo, J. Kozlitina, L. Valenti

**Affiliations:** ^1^ Internal Medicine and Metabolic Diseases Fondazione IRCCS Ca' Granda Ospedale Policlinico Milano Milan Italy; ^2^ Department of Molecular Genetics University of Texas Southwestern Medical Center Dallas TX USA; ^3^ Bioinformatic unit Istituto Nazionale Genetica Molecolare Milan Italy; ^4^ Department of Molecular and Clinical Medicine University of Gothenburg Gothenburg Sweden; ^5^ Department of Gastroenterology Università di Palermo Palermo Italy; ^6^ Department of Surgery Fondazione IRCCS Ca' Granda Ospedale Policlinico Milano Milan Italy; ^7^ Department of Pathology Fondazione IRCCS Ca' Granda Ospedale Policlinico Milano Milan Italy; ^8^ Department of Gastroenterology University of Eastern Finland Kuopio University Hospital Kuopio Finland; ^9^ Department of Medicine University of Eastern Finland Kuopio University Hospital Kuopio Finland; ^10^ Clinical Nutrition and Obesity Center Kuopio University Hospital Kuopio Finland; ^11^ Department of Public Health and Clinical Nutrition University of Eastern Finland Kuopio Finland; ^12^ Institute of Medicine Sahlgrenska Academy University of Gothenburg Gothenburg Sweden; ^13^ Department of Pathophysiology and Transplantation Università degli Studi di Milano Milan Italy; ^14^ Clinical Nutrition Unit Department of Medical and Surgical Sciences University Magna Graecia Catanzaro Italy; ^15^ Cardiology Department Sahlgrenska University Hospital Gothenburg Sweden; ^16^ McDermott Center for Human Growth and Development University of Texas Southwestern Medical Center Dallas TX USA

**Keywords:** fibrosis, genetics, insulin resistance, mendelian randomization, nonalcoholic fatty liver disease, type 2 diabetes

## Abstract

**Background and Aims:**

Nonalcoholic fatty liver disease is epidemiologically associated with hepatic and metabolic disorders. The aim of this study was to examine whether hepatic fat accumulation has a causal role in determining liver damage and insulin resistance.

**Methods:**

We performed a Mendelian randomization analysis using risk alleles in *PNPLA3*,* TM6SF2*,* GCKR* and *MBOAT7,* and a polygenic risk score for hepatic fat, as instruments. We evaluated complementary cohorts of at‐risk individuals and individuals from the general population: 1515 from the liver biopsy cohort (LBC), 3329 from the Swedish Obese Subjects Study (SOS) and 4570 from the population‐based Dallas Heart Study (DHS).

**Results:**

Hepatic fat was epidemiologically associated with liver damage, insulin resistance, dyslipidemia and hypertension. The impact of genetic variants on liver damage was proportional to their effect on hepatic fat accumulation. Genetically determined hepatic fat was associated with aminotransferases, and with inflammation, ballooning and fibrosis in the LBC. Furthermore, in the LBC, the causal association between hepatic fat and fibrosis was independent of disease activity, suggesting that a causal effect of long‐term liver fat accumulation on liver disease is independent of inflammation. Genetically determined hepatic steatosis was associated with insulin resistance in the LBC and SOS. However, this association was dependent on liver damage severity. Genetically determined hepatic steatosis was associated with liver fibrosis/cirrhosis and with a small increase in risk of type 2 diabetes in publicly available databases.

**Conclusion:**

These data suggest that long‐term hepatic fat accumulation plays a causal role in the development of chronic liver disease.

## Introduction

Nonalcoholic fatty liver disease (NAFLD) is defined by the accumulation of fat in the liver, in the absence of excessive alcohol consumption. NAFLD encompasses a spectrum of conditions ranging from simple accumulation of excess fat (steatosis) to hepatic inflammation (nonalcoholic steatohepatitis or NASH) and fibrosis. Obesity and insulin resistance are major risk factors for NAFLD 1, 2, 3. Concurrent with the increased prevalence of obesity, NAFLD has emerged as the most frequent liver disorder worldwide, affecting as many as 30% of adults in industrialized countries 4, 5.

Although simple steatosis is generally considered benign, NAFLD is associated with an increased risk of end‐stage liver disease, as well as a range of extrahepatic metabolic disorders, including insulin resistance, type 2 diabetes (T2D), dyslipidemia and hypertension. However, the extent to which these associations reflect a causal effect of hepatic fat accumulation remains unclear. Associations observed in epidemiological studies may be influenced by confounding factors (e.g. diet, physical activity and microbiota) and/or reverse causation. Furthermore, hepatic fat accumulation tends to decrease with the progression of fibrosis (‘burnt‐out nonalcoholic steatohepatitis’) 6, potentially limiting the ability of cross‐sectional association studies to reliably evaluate the correlates of hepatic fat in patients with severe disease 7. Weight‐loss interventions aimed at reducing liver fat lead to improved metabolic outcomes 8. However, it is unclear whether these changes are a direct result of the reduction in liver fat or a consequence of the improvement in extrahepatic insulin resistance or improved lifestyle 3.

Mendelian randomization is an epidemiological method that avoids confounding and reverse causation, by using genetic variation as an instrument to establish the causal role of modifiable risk factors in disease 9, 10. The method relies on the assumption that as an individual's genotype is determined randomly at conception, is not related to lifestyle and other potential confounding factors, and thus can serve as an unconfounded and lifelong proxy for an exposure of interest (e.g. hepatic fat content). As our knowledge of common genetic variation governing variability in quantitative traits has improved over the past decade as a result of genomewide association studies (GWAS), Mendelian randomization has gained widespread use as a method of testing causal effects of modifiable risk factors in situations where randomized controlled trials are not feasible or ethical 11. For example, the approach has been used successfully to assess the causal relationship of different lipoprotein fractions with the risk of cardiovascular disease 11. Previous genomewide association studies have identified several genetic risk factors for NAFLD, including variants in *PNPLA3* 12, 13, *TM6SF2* 14, 15, *GCKR* 13, *MBOAT7* 16, 17 and possibly *LYPLAL1* 13, which can be used as instruments to assess the causal effect of hepatic fat on progressive liver disease. Previous studies have shown most of these variants to be associated with NAFLD development and severity 12, 13, 14, 17, 18. However, whether the magnitude of association of these genetic variants with progressive liver disease is concordant with an increase in risk predicted from their effect on hepatic fat accumulation has not been tested so far. Furthermore, whether genetically determined hepatic fat influences insulin resistance remains a controversial question 3, 19, 20.

Here, we leveraged a Mendelian randomization approach to examine whether hepatic fat causally determines liver damage and metabolic comorbidities. To this end, we evaluated complementary cohorts of individuals at risk of progressive NAFLD due to suspected NASH in the Liver Biopsy Cohort (LBC), or to severe obesity in the Swedish Obese Subjects Study (SOS), and individuals from the population‐based Dallas Heart Study (DHS). We sequentially tested whether the steatosis‐associated variants, individually or combined into a genetic risk score (GRS), were associated with liver damage (histological features of NAFLD, aminotransferases and noninvasive fibrosis scores) and clinical parameters epidemiologically associated with insulin resistance and NAFLD (presence of arterial hypertension, T2D, homoeostasis metabolic assessment‐insulin resistance index – HOMA‐IR, and circulating HDL cholesterol).

## Materials and methods

### Study cohorts

Part of the LBC has been previously described 17, 21. Briefly, a total of 1515 adult European individuals who underwent liver biopsy for suspected NASH or severe obesity were consecutively enrolled. Individuals with increased alcohol intake (men, >30 g day^−1^; women, >20 g day^−1^), use of steatogenic medications or other liver diseases were excluded. Demographic and clinical features of patients included in the LBC were evaluated at the tertiary referral centres at the time of liver biopsy. The SOS is a prospective study comparing the effects of bariatric surgery and usual care in severely obese individuals in Sweden 22. A total of 3329 subjects, who had complete baseline metabolic characterization and were successfully genotyped, were included. The DHS is a multiethnic population‐based probability sample of Dallas County residents 23. The present investigation included a total of 4570 individuals, who had provided blood samples for genetic and laboratory analysis. Of these, 2736 individuals had available measures of liver fat with proton magnetic resonance spectroscopy (^1^H‐MRS) 1.

**Figure 1:**
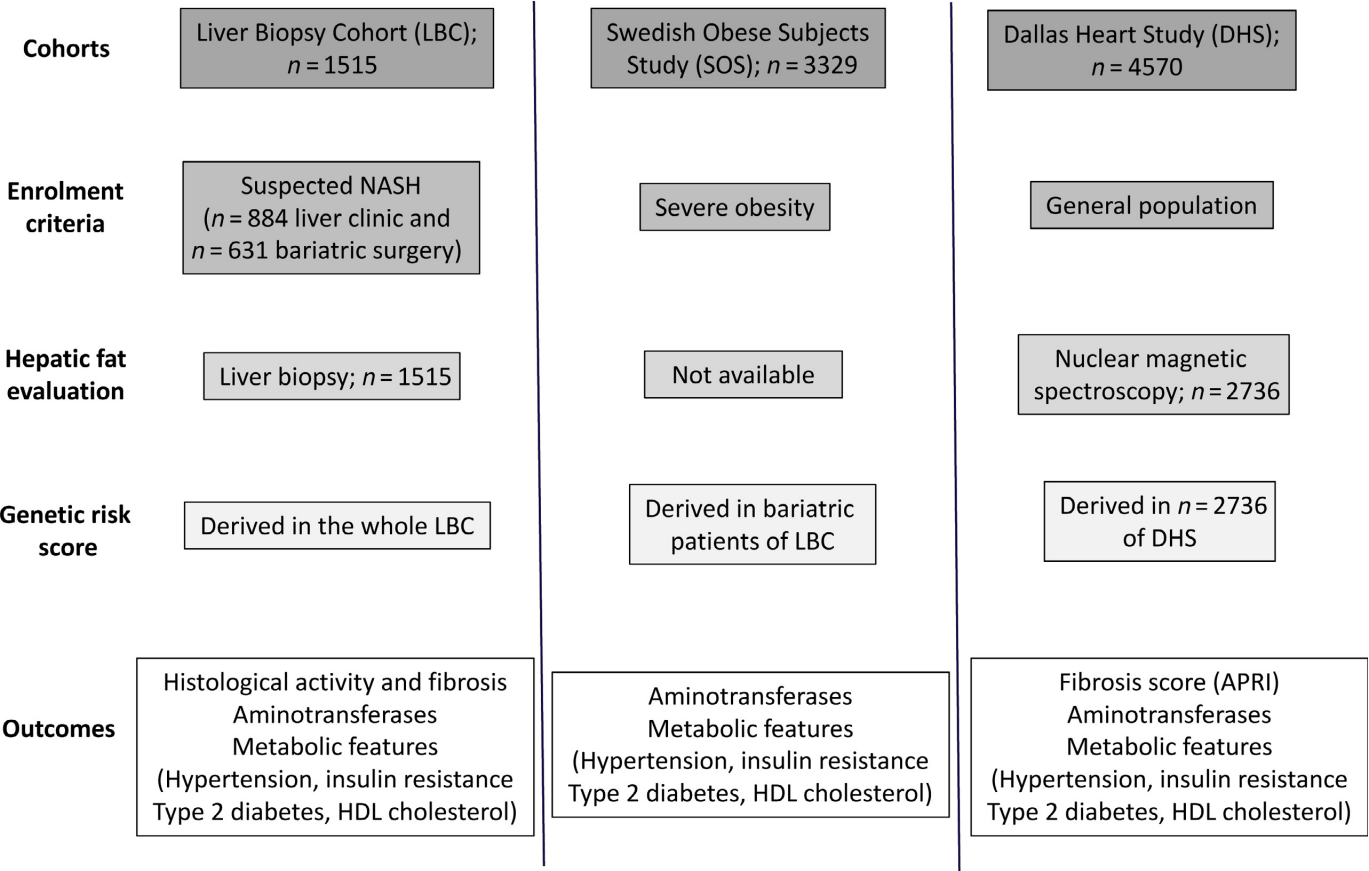
Study cohorts and outcomes. NASH, nonalcoholic steatohepatitis.

All studies were approved by the competent ethical committees, and all subjects gave written or oral informed consent. The study cohort characteristics and outcomes are summarized in Fig. 1, clinical features of individuals evaluated in the study are presented in Table 1, and additional details concerning the study design are provided in Supporting information (Data S1) and in Figure S1.

**Table 1:** Clinical features of the cohorts of individuals included in the study

	Liver biopsy cohort (*N* = 1515)	Swedish Obese Subjects Study (*N* = 3329)	Dallas Heart Study (*N* = 4570)
Age, years	43 ± 16	48 ± 6	45 ± 11
Sex, female	722 (48)	2338 (70)	2608 (57)
Ethnicity			
European/European descent	1515 (100)	NA^a^	1351 (30)
African American	–	–	2355 (51)
Hispanic	–	–	743 (16)
Recruitment liver disease/morbid obesity^b^	884 (58)/631 (42)	0/3329 (100)	–
ALT, IU L^−1^	42 (23–69)	30 (22–42)	19 (14–27)
AST, IU L^−1^	28 (20–42)	22 (17–28)	21 (17–26)
Statin use, yes	127 (8)	–	390 (9)
Hepatic triglyceride content, %	–	–	3.5 (1.9–7.0)
			{*N* = 2736}
Fatty liver, yes^c^	1346 (90)	–	880 (32)
			{*N* = 2736}
Fibrosis F3‐F4, yes	346 (23)	–	–
BMI, kg m^−2^	32.1 (27–40)	41.0 (38–44)	29.4 (25–34)
Hypertension, yes	459 (36)	2288 (69)	1496 (36)
T2D, yes	400 (26)	501 (15)	561 (12)
Fasting glucose, mg dL^−1^ d	95 (86–110)	81 (72–94)	93 (85–102)
			{*N* = 4474}
Fasting insulin, IU mL^−1^	15 (9–21)	17 (12–24)	13 (8–20)
	{*N* = 1082}		{*N* = 4018}
HOMA‐IR, U	3.5 (2.2–5.3)	3.5 (2.3–5.5)	3.0 (1.7–5.0)
	{*N* = 1082}		{*N* = 4018}
Total cholesterol, mg dL^−1^ e	193 ± 46	220 ± 43	183 ± 40
HDL cholesterol, mg dL^−1^ e	46 ± 8	50 ± 11	51 ± 15
		{*N* = 3203}	
Triglycerides, mg dL^−1^ f	124 (88–177)	159 (115–221)	97 (68–145)
*N*. of risk alleles	3 (2–4)	2 (1–3)	2 (1–3)
GRS	0.53 (0.47–0.53)	0.43 (0.37–0.48)	0.13 (0.06–0.34)

Values are reported as mean ± SD, number (%), or median (IQR), as appropriate. *N*, number; NA, not assessed; BMI, body mass index; T2D, type 2 diabetes; HOMA‐IR, homoeostasis model assessment‐insulin resistance index; HDL, high‐density lipoprotein; ALT, alanine aminotransferases; AST, aspartate aminotransferases; GRS, genetic risk score. Numbers in curly brackets indicate numbers of individuals with available data for each phenotype. ^a^In the SOS, information about ethnicity was not available, but most participants are presumed of European descent; ^b^Defined in the presence of BMI ≥ 40 kg m^−2^ or ≥35 kg m^−2^ in patients with metabolic comorbidities (type 2 diabetes, dyslipidemia or arterial hypertension). ^c^In DHS, NAFLD (steatosis) was defined as hepatic triglyceride content (HTGC) >5.5%. ^d^Multiply by *0.055 ^e^0.026 ^f^0.011 to obtain mmol L^−1^.

### Biochemical and clinical end‐points

Hypertension was defined as systolic blood pressure ≥140 mm Hg or diastolic blood pressure ≥90 mm Hg, or self‐reported antihypertensive treatment. T2D was defined as fasting blood glucose level ≥126 mg dL^−1^, nonfasting glucose ≥200 mg dL^−1^, HbA1c ≥6.5% or use of glucose‐lowering medication. HOMA‐IR was calculated from fasting levels of glucose and insulin 24. Plasma levels of lipids and lipoproteins were measured by standard enzymatic assays. As most of the risk variants included in the genetic risk score have been shown to have pleiotropic effects on circulating cholesterol and triglycerides 18, they were not regarded as valid instruments for testing the causal effect of hepatic fat on these outcomes. Therefore, only HDL levels were considered as an outcome in this study.

In the LBC, steatosis, disease activity and fibrosis were assessed according to the NAFLD clinical research network criteria 25. Briefly, steatosis was scored (on a scale from 0 to 3) according to the percentage of affected hepatocytes (0: <5%, 1: 5–33%, 2: 34–66%, 3: 67–100%), and hepatocellular ballooning (0–2) and lobular necroinflammation (0–3) were also recorded. Fibrosis stage was staged (from 0, no fibrosis to 4, cirrhosis) to evaluate disease progression 25. The scoring of liver biopsies was performed by independent pathologists unaware of the clinical history and genotype of the patients 21. The concordance between pathologists within this cohort was very good for fibrosis and good for steatosis with a coefficient of interobserver agreement for fibrosis stage and steatosis grade of 0.89 and 0.76, respectively 26.

In the DHS, we used the APRI score (AST/platelets × 100) as a noninvasive marker of fibrosis 27, because it showed the strongest association with hepatic fat compared to other indices (Table S1).

### Genotyping

To test the causal role of hepatic fat, we selected genetic variants with validated effects on hepatic fat accumulation and/or NAFLD severity (in LBC, DHS and previous GWAS 12, 13, 14, 16, 28) and with experimental data supporting the causality of the association, as instruments.

Therefore, the LBC and SOS cohorts were genotyped for rs738409 C>G (*PNPLA3* I148M), rs58542926 C>T (*TM6SF2* E167K), rs1260326 C>T (*GCKR* P446L) and rs641738 C>T in the *MBOAT7* locus, as previously described 17, 21. The genotyping was performed in duplicate by TaqMan 5′‐nuclease assays in the LBC and SOS (Life Technologies, Carlsbad, CA, USA). The duplicate genotype concordance rate was 100%. In the DHS, genotyping was performed using Illumina Human Exome BeadChip, as described 12. A subset of DHS participants (*n* = 3300) was previously genotyped for *LYPLAL1* rs12137855 using TaqMan assays. As the genotypes for this variant were not available in the LBC and SOS, we did not include this variant in our primary analysis but considered it as part of sensitivity analysis (see Supporting information, Data S2).

### Evaluation of associations of NAFLD‐associated variants with T2D and liver‐related end‐points in GWAS data

We looked up the associations with T2D of the NAFLD‐associated genetic variants in publicly available databases from T2D GWAS 29, 30. Data were downloaded from the Diagram Consortium at http://diagram-consortium.org/downloads.html, and in the case of *TM6SF2* rs58542926 (E167K), extracted directly from Fuchsberger *et al*. 30. As *GCKR* rs1260326 (P446L) has likely pleiotropic effects on glucose metabolism, we reported the effect but did not consider it as a proxy for hepatic fat when evaluating T2D risk in a Mendelian randomization context.

Two‐sample Mendelian randomization analysis was performed using the ‘MendelianRandomization’ package in R. The per‐allele associations of the *PNPLA3*,* TM6SF2* and *MBOAT7* variants with hepatic fat content in the DHS were entered as exposures, and the GWAS associations with T2D were used as outcomes. A summary estimate was calculated using the inverse variance weighted method. As described above, *GCKR* rs1260326 (P446L) was not included in this analysis due to the strong pleiotropic effects of this SNP on plasma glucose levels.

Associations with ICD‐code defined liver end‐points were extracted from http://geneatlas.roslin.ed.ac.uk/, a publically available database of genetic associations in the UK Biobank (*n* = 408 455). Beta coefficients and *P*‐values were extracted for associations with ICD‐10 K76 (‘other diseases of liver’) and K74 (‘fibrosis and cirrhosis of liver’).

### Statistical analysis

We used four established risk variants for hepatic fat accumulation 17, 31 as instruments in Mendelian randomization analysis. To increase power, we also created a genetic risk score (GRS) for hepatic fat accumulation composed of these variants. The association of each genetic variant with hepatic steatosis was estimated using linear regression models in all individuals with available measures of hepatic steatosis (1515 in LBC and 2736 in DHS). GRS was then calculated across all three cohorts by summing the number of steatosis predisposing alleles, weighted by their effect size (beta coefficient) on steatosis. Weights derived from severely obese individuals in the LBC were used to calculate the GRS in the SOS cohort, due to similar ethnic and clinical make‐up of the participants. The GRS was strongly correlated with steatosis, explaining 7.0% and 3.5% of its variability in the LBC and DHS, respectively (Table S2). As steatosis was graded on an ordinal categorical scale in the LBC, we also performed a sensitivity analysis, using ordinal regression models to assess the relationship of genetic variants with steatosis grade. GRS constructed based on ordinal regression models had very similar associations with liver damage and metabolic outcomes. Therefore, we decided to use the GRS estimated from linear regression models (in both cohorts) for our primary analyses.

Observational associations of hepatic steatosis with NAFLD and NAFLD‐associated metabolic traits were assessed using linear and logistic regression models, with steatosis grade (LBC) or hepatic fat content (DHS) as an explanatory variable, and each trait as the outcome. The causal effect of hepatic steatosis on NAFLD and metabolic traits was estimated using the Wald (ratio) method, with standard errors approximated by the delta method 32. All models were adjusted for age, sex, recruitment centre (in the LBC), ethnicity (in DHS), body mass index (BMI) and statin use. All estimates are standardized. The reported observational estimates are for a 1‐SD unit increase in hepatic fat. The causal estimates are for a 1‐SD unit increase in genetically determined hepatic fat content. We conducted sensitivity analyses, using alternative GRS, calculated by excluding one genetic variant at a time, and using an unweighted risk score.

Statistical analyses were carried out using R statistical analysis software version 3.3.2 (http://www.R-project.org/). *P*‐values < 0.05 were considered statistically significant.

## Results

### Observational association of hepatic fat with liver damage and metabolic traits

In the observational analysis, hepatic fat content was strongly associated with aminotransferases (*P* < 10^−16^, Table 2), in two of the study cohorts with available measures of hepatic fat (LBC and DHS). Further, it was associated with histological necroinflammation (*P* < 10^−115^), ballooning (*P* < 10^−48^) and fibrosis stage (*P* < 10^−50^) in the LBC, and with the APRI fibrosis score in the DHS (*P* = 9.8 × 10^−5^). In both cohorts, hepatic fat was also associated with the prevalence of hypertension, T2D, increased insulin resistance (HOMA‐IR) and with lower HDL (all *P* < 0.05).

**Table 2:** Observational association of study outcomes with histological steatosis severity in the liver biopsy cohort (LBC; *n* = 1515 at risk of NASH) and hepatic TG content in the Dallas Heart Study (DHS; *n* = 2736 from the general population)

	Liver biopsy cohort (*N* = 1515)			Dallas Heart Study (*N* = 2736)		
	β	95% c.i.	*P* value	β	95% c.i.	*P* value
ALT, IU L^−1^	+0.34	(0.30–0.38)	9.0 × 10^−54^	+0.25	(0.22–0.29)	2.0 × 10^−40^
AST, IU L^−1^	+0.29	(0.25–0.34)	4.0 × 10^−35^	+0.17	(0.13–0.21)	6.6 × 10^−17^
Necroinflammation	+0.53	(0.49–0.58)	5.0 × 10^−115^	–	–	–
Ballooning	+0.33	(0.29–0.38)	7.1 × 10^−49^	–	–	–
Fibrosis stage	+0.35	(0.30–0.39)	2.1 × 10^−51^	–	–	–
APRI score^a^	–	–	–	+0.09	(0.04–0.13)	9.8 × 10^−5^
Hypertension, yes	+0.13	(0.07–0.18)	1.0 × 10^−5^	+0.17	(0.06–0.27)	0.0018
T2D, yes	+0.12	(0.08–0.17)	5.8 × 10^−7^	+0.45	(0.30–0.59)	2.6 × 10^−9^
HOMA‐IR	+0.25	(0.20–0.31)	1.5 × 10^−22^	+0.29	(0.26–0.33)	1.3 × 10^−60^
HDL	−0.06	(−0.11 to −0.01)	0.011	−0.15	(−0.19 to −0.12)	5.5 × 10^−15^

Adjusted standardized beta coefficients and (95% c.i.) are reported. Coefficients were adjusted for age, sex, BMI, use of statins, recruitment centre in the LBC or ethnicity in the DHS. The regression coefficients are expressed in unit of SD of feature per unit increment in histological steatosis grade (which was approximated to a continuous trait and normalized) in the LBC or a 1 SD change in hepatic triglyceride content in the DHS. For binary outcomes, the relationship was assessed using logistic regression, with beta coefficients representing the log of the odds of the outcome per a one‐unit increment in steatosis grade or a 1 SD change in hepatic fat content. BMI, body mass index; T2D, type 2 diabetes; HOMA‐IR, homoeostasis model assessment‐insulin resistance index; HDL, high‐density lipoprotein cholesterol; ALT, alanine aminotransferases; AST, aspartate aminotransferases. After correction for multiple comparisons, *P* < 0.0065 and *P* < 0.0071 are considered statistically significant in the LBC and DHS, respectively. ^a^Available for *N* = 1933 in DHS.

### Association of genetic variants with hepatic fat, liver damage and metabolic traits

The frequency distribution of the number of risk alleles carried by each individual in the three cohorts is shown in Figure S2. The impact of the individual risk variants on hepatic fat, liver damage and metabolic traits is presented in Table S3. As previously reported, in the LBC and DHS, *PNPLA3* rs738409 (I148M) was the strongest genetic determinant of hepatic fat, followed by *TM6SF2* rs58542926 (E167K), while *GCKR* rs1260326 (P446L) and *MBOAT7* rs641738 had smaller but significant effects. Of the four individual variants, *PNPLA3* rs738409 (I148M) was also associated with higher liver enzymes in all three cohorts. All four gene variants were associated with the full spectrum of liver damage, related to NAFLD, in the LBC.

**Figure 2:**
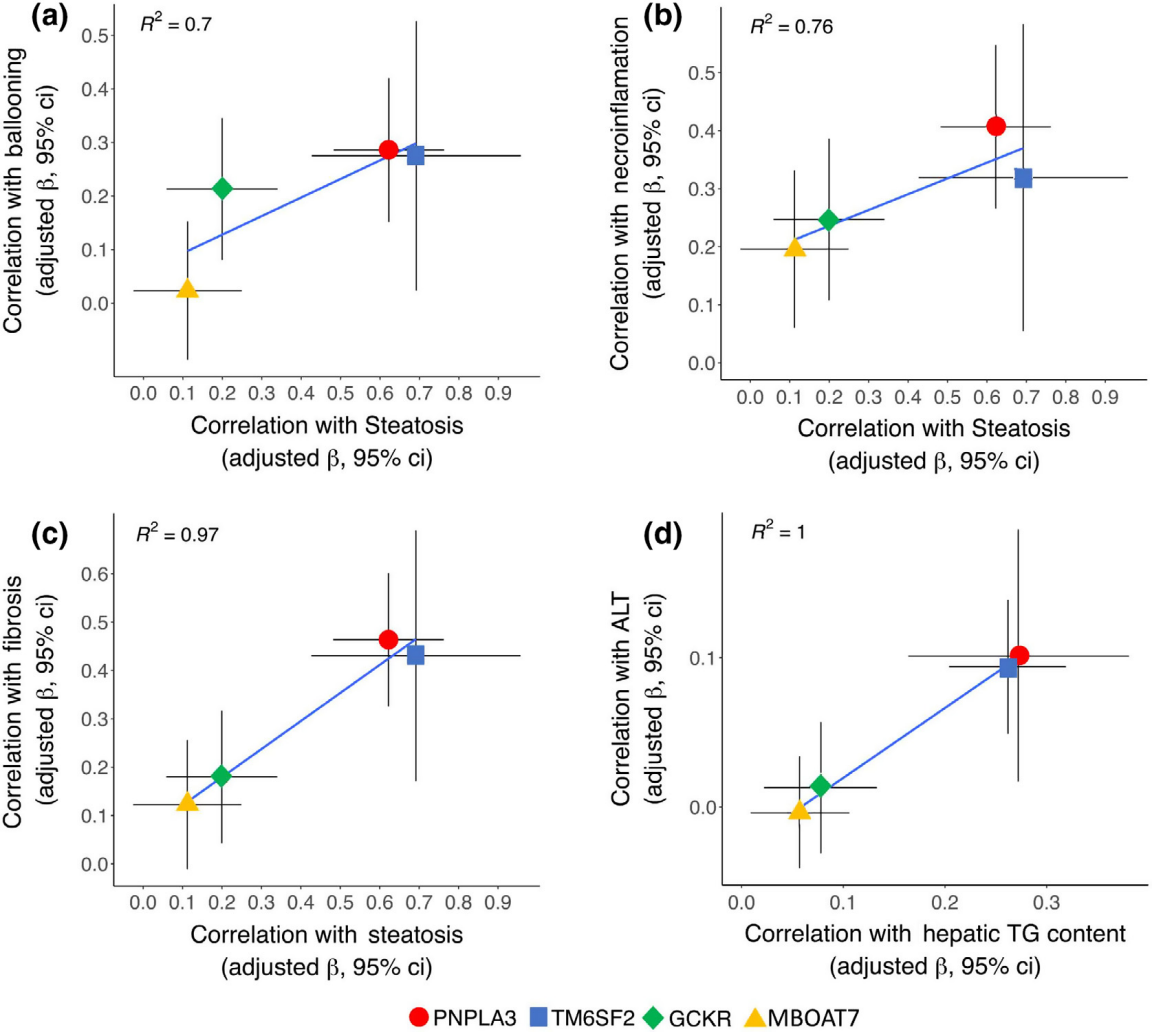
Comparison of the impact of risk variants: *PNPLA3* I148M (rs738409), *TM6SF2* E167K (rs58542926), *GCKR* P446L (rs1260326) and *MBOAT7* rs641738 on hepatic fat vs. liver damage. Panel a: histological steatosis vs. ballooning in the LBC (*R* ^2^ = 0.70). b: histological steatosis vs. necroinflammation in the LBC (*R* ^2^ = 0.76). c: histological steatosis vs. fibrosis in the LBC (*R* ^2^ = 0.97). d: hepatic fat content vs. serum ALT levels in the DHS (*R* ^2^ = 1). Beta coefficients and 95% confidence intervals are shown for each variant. Correction added on 16 January 2018 after first online publication: In Figure 2B and D, the color labels for PNPLA3 and TM6SF2 have been interchanged for clarity]

The impact of genetic risk variants on liver damage was proportional to their effect on hepatic fat accumulation (Fig. 2), consistent with the notion that their impact on liver damage is mainly mediated by increased liver fat and not by other pleiotropic effects. In a sensitivity analysis using ordinal regression models, we confirmed a close correlation between the impact of genetic variants on steatosis and that on fibrosis in the LBC (Figure S3).

The *PNPLA3* rs738409 (I148M) variant was associated with increased HOMA‐IR in the LBC and SOS cohorts but not in the DHS (Table S3).

### Causal effect of hepatic fat on liver damage and metabolic traits

**Figure 3:**
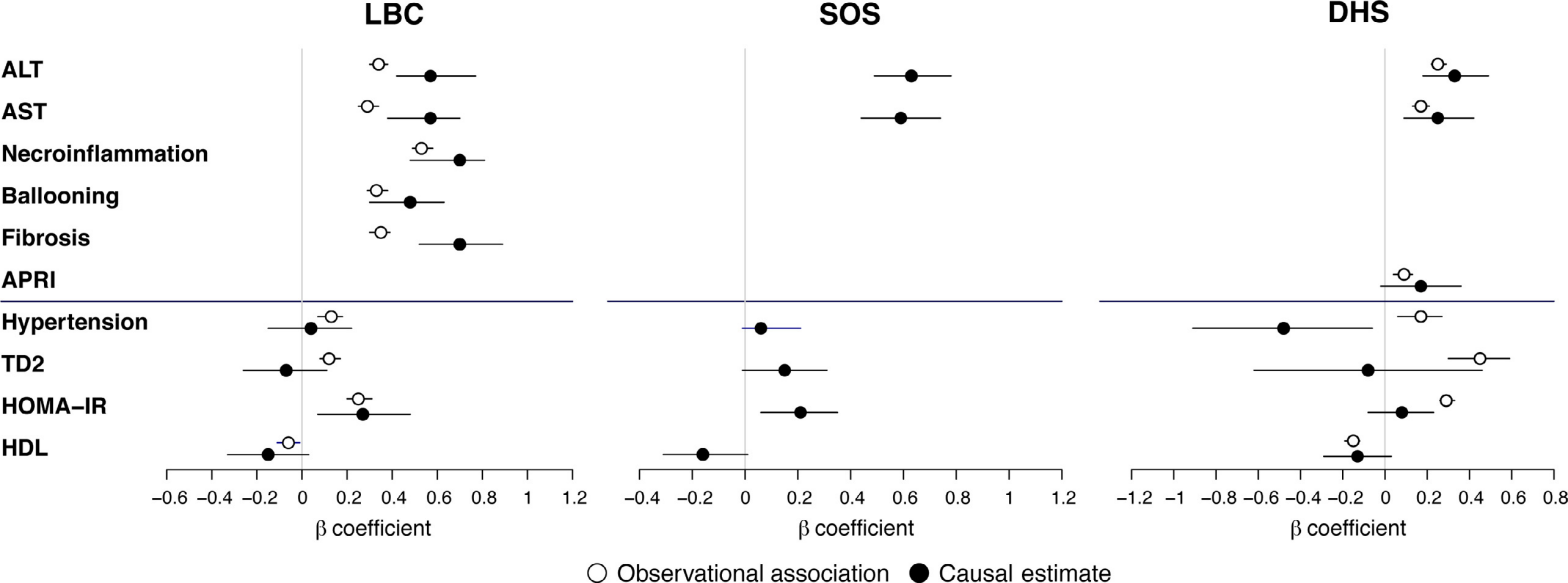
Comparison of the epidemiological association of observed hepatic fat with NAFLD features (open circles) with the causal association of hepatic fat with NAFLD‐related features (filled circles) in the LBC (*n* = 1515, panel a), the SOS (*n* = 3329, panel b) and the DHS (*n* = 2736, panel c). T2D, type 2 diabetes; HOMA‐IR, homoeostasis model assessment‐insulin resistance index; HDL, high‐density lipoprotein; ALT, alanine aminotransferases; AST, aspartate aminotransferases. Results were adjusted for age, sex, BMI, modality of recruitment in the LBC and ethnicity in the DHS, use of statins and (for the metabolic parameters) the severity of liver fibrosis. Estimates are beta coefficients, and error bars are 95% confidence intervals.

The estimated causal effects of hepatic fat on histological and metabolic traits associated with NAFLD are shown in Table 3 and in Fig. 3. The GRS was not associated with age, sex and anthropometric traits (not shown). A genetically determined increase in hepatic fat was associated with elevated aminotransferases in all three cohorts (*P* < 10^−9^ in the LBC, *P* < 10^−14^ in the SOS and *P* < 10^−3^ in the DHS) and with histological features of NAFLD‐related liver damage in the LBC, including necroinflammation and ballooning (*P* < 10^−16^ and *P* < 10^−9^, respectively), as well as fibrosis stage (*P* < 10^−12^). Of note, in the LBC, genetically predicted steatosis had a larger impact on fibrosis than the observed histological steatosis. Finally, the causal association between hepatic fat and fibrosis was independent of necroinflammation and hepatocellular ballooning (β = +0.37, 95% c.i. 0.21–0·52; *P* = 5.2 × 10^−6^). In a sensitivity analysis that used two‐stage least squares regression (rather than the ratio method) to calculate causal estimates, the results did not change substantially (Data S1 and Table S4), except that genetically determined hepatic fat was additionally associated with fibrosis (as estimated by the APRI score) in the DHS (*P* < 0.05). These results suggest that hepatic fat accumulation causally promotes the full spectrum of liver damage associated with NAFLD.

**Table 3:** Causal association of genetically determined hepatic fat (GRS, genetic risk score) with liver damage and clinical parameters epidemiologically associated with NAFLD in the three study cohorts

Outcome	Liver biopsy cohort (*N* = 1515)			Swedish Obese Subjects Study (*N* = 3329)			Dallas Heart Study (*N* = 4455)^a^		
	β	95% c.i.	*P* value				β	95% c.i.	*P* value
ALT	+0.57	(0.42–0.77)	2.8 × 10^−11^	+0.63	(0.490.78)	1.4 × 10^−17^	+0.33	(0.18–0.49)	2.8 × 10^−5^
AST	+0.57	(0.38–0.70)	8.0 × 10^−10^	+0.59	(0.44–0.74)	1.9 × 10^−14^	+0.25	(0.09–0.41)	0.0019
Necroinflammation	+0.70	(0.48–0.81)	6.6 × 10^−12^	–	–	–	–	–	–
Ballooning	+0.48	(0.30–0.63)	2.2 × 10^−7^	–	–	–	–	–	–
Fibrosis	+0.70	(0.52–0.89)	1.3 × 10^−14^	–	–	–	–	–	–
APRI score^b^	–	–	–	–	–	–	+0.17	(−0.01 to 0.35)	0.061
Hypertension	+0.04	(−0.15 to 0.22)	0.78	+0.06	(−0.01 to 0.21)	0.47	−0.48	(−0.91 to −0.05)	0.028
T2D	−0.07	(−0.26 to 0.11)	0.42	+0.15	(−0.01 to 0.31)	0.057	−0.08	(−0.62 to 0.46)	0.77
HOMA‐IR	+0.27	(0.07–0.48)	0.0089	+0.21	(0.06–0.35)	0.006	+0.08	(−0.07 to 0.22)	0.30
HDL	−0.15	(−0.33 to 0.03)	0.10	−0.16	(−0.31 to 0.01)	0.039	−0.13	(−0.28 to 0.02)	0.083

Adjusted standardized beta coefficients and (95% c.i.) are reported. Coefficients were adjusted for age, sex, BMI, use of statins, recruitment centre in the LBC or ethnicity in the DHS. The regression coefficients are expressed in unit of SD of feature per unit increment in GRS. For binary outcomes, the relationship was assessed using logistic regression, with beta coefficients representing the log of the odds of the outcome per a one‐unit increment in GRS. BMI, body mass index; T2D, type 2 diabetes; HOMA‐IR, homoeostasis model assessment‐insulin resistance index; HDL, high‐density lipoprotein; LDL, low‐density lipoprotein; ALT, alanine aminotransferases; AST, aspartate aminotransferases; HF, hepatic fat, that is normalized histological steatosis of hepatic triglycerides content. After correction for multiple comparisons, *P* < 0.0065, *P* < 0.008 and *P* < 0.0071 are considered statistically significant in the LBC, SOS and DHS, respectively. ^a^All individuals with available genotype, phenotype and covariate data (up to *N* = 4455) were included. ^b^Available for *N* = 3133 in DHS.

In the LBC, genetically determined hepatic fat was associated with HOMA‐IR, to an extent similar to that of observed steatosis, suggesting that hepatic fat causes insulin resistance, consistent with the reported epidemiological association (Table 3 and Fig. 3). The effect of steatosis on HOMA‐IR became more marked after excluding patients with T2D (β = +0.41, 95% c.i. 0.15–0.64; *P* = 0.00078), but it was attenuated after correction for fibrosis (β = +0.10, 95% c.i. −0.10–0.33; *P* = 0.26). Similarly, in the SOS, genetically determined hepatic fat was causally associated with HOMA‐IR (β = +0.21; 95% c.i. 0.06–0.35; Table 3 and Fig. 3), but the association was lost after correction for serum ALT, used as a measure of liver damage (β = +0.10; 95% c.i. −0.05–0.24; *P* = 0.20). We did not detect a significant effect of genetically determined hepatic fat on insulin resistance in the population‐based DHS (Table 3).

To validate the causal association between hepatic fat and insulin resistance, we extracted results from the largest publicly available GWAS on T2D (Table 4). The risk alleles of the two strongest steatogenic variants, *PNPLA3* rs738409 (I148M) and *TM6SF2* rs58542926 (E167K), were associated with a small increase in the risk of T2D (allelic ORs 1.04, 95% c.i. 1.01–1.07 and 1.14, 95% c.i. 1.10–1.19, respectively). The association of the *MBOAT7* variant was directionally concordant but not significant (OR 1.02, 95% c.i. 0.99–1.05; *P* = 0.19). The major (P) allele of *GCKR* rs1260326 P446L (which reduces the risk of NAFLD) was associated with a modestly increased risk of T2D (OR 1.06, 95% c.i. 1.03–1.09). However, GCKR rs1260326 P446L is known to associate strongly with plasma glucose levels, a pleiotropic association that makes it problematic to use this variant as a proxy for hepatic fat in a Mendelian randomization context with T2D as an outcome.

**Table 4:** Association of NAFLD‐variants with type 2 diabetes in published GWAS

Gene	SNP	Effect allele	other allele	T2D OR	95% c.i.	*P* value	*N*	Data source
*PNPLA3*	rs738409	G (minor)	C	1.04	1.01–1.07	0.0045	100 323	diagram.mega‐meta
*TM6SF2*	rs58542926	T (minor)	C	1.14	1.10–1.19	3.2 × 10^−10^	92 794	Fuchsberger 2016 30
*MBOAT7*	rs641738	T (minor)	C	1.02	0.99–1.05	0.19	76 306	diagram.mega‐meta
*GCKR*	rs1260326	C (major)^a^	T	1.06	1.03–1.09	3.4 × 10^−5^	100 584	diagram.mega‐meta

Data were downloaded from http://diagram-consortium.org/downloads.html or extracted from Fuchsberger *et al*. 30. ^a^The effect allele in *GCKR* is the major allele, encoding *GCKR* 446P. The 446P‐allele causes a relative gain‐of‐function compared to the L‐allele, leading to less hepatic phosphorylation of glucose, increased blood glucose and thus an increased risk of type 2 diabetes (T2D). Due to these strong pleiotropic effects of GCKR on glucose metabolism, it is problematic to use this variant as a proxy for NAFLD in Mendelian randomization analyses with T2D as an outcome.

In two‐sample Mendelian randomization analysis using the *PNPLA3*,* TM6SF2* and *MBOAT7* variants as instruments for hepatic fat, the causal OR for risk of T2D for a 1 standardized unit increase in genetically determined hepatic fat was 1.31 (95% c.i., 1.20–1.43; *P* = 1.2 x 10^−9^).

### Sensitivity analyses

To rule out the possibility that the association of the GRS with NAFLD‐associated traits was biased by the specific mechanism of action of single genetic risk variants, we conducted sensitivity analyses using alternative GRS, calculated by excluding a single risk variant at a time (Table S2). The results evaluating the association of alternative GRS with NAFLD features are shown in Table S5.

All GRS were associated with hepatic fat, although, as expected, those excluding *PNPLA3* rs738409 (I148M) were less robustly associated than the other scores, across all cohorts. The associations between alternative GRS and NAFLD‐associated traits were generally consistent with those for the full score, based on four variants. Importantly, most alternative models confirmed a causal association of hepatic fat with liver enzymes and histological liver damage. The only exception was that the associations with aminotransferases in the DHS were no longer statistically significant when excluding the *PNPLA3* variant, likely due to the lack of power (Table S5). In the LBC, we also confirmed a trend for association of the alternative GRS with increased insulin resistance.

To confirm the association of the steatogenic variants with liver disease in a separate cohort, we performed a lookup in a publically available database of genetic associations in 408 455 participants from UK Biobank (as reported in the Methods and in Supporting information, Data S1). The steatogenic alleles in *PNPLA3*,* TM6SF2*,* GCKR* and *MBOAT7* all associated with increased risk of ICD‐defined ‘other diseases of liver’ (K76), which includes NAFLD (*n* = 408 455, *P* = 0.0075–2 × 10^−12^, Table S6 and Figure S4). Variants in *PNPLA3*,* TM6SF2* and *MBOAT7* associated with increased risk of ‘fibrosis and cirrhosis of liver’ (K74; *P* = 0.002–3 × 10^−6^, Table S6). In two‐sample Mendelian randomization analysis, a standardized 1‐unit increase in genetically determined hepatic fat content was associated with an increased risk of ‘other diseases of liver’ (*P* = 5 × 10^−19^), and with ‘fibrosis and cirrhosis of liver’ (*P* = 9 × 10^−11^).

Additional sensitivity analyses, evaluating an alternative GRS including the *LYPLAL1* rs12137855 variant, using beta coefficients derived from external cohorts, and showing the results in patients stratified according to modality of recruitment and ethnicity are reported in Supporting information.

## Discussion

**Figure 4:**
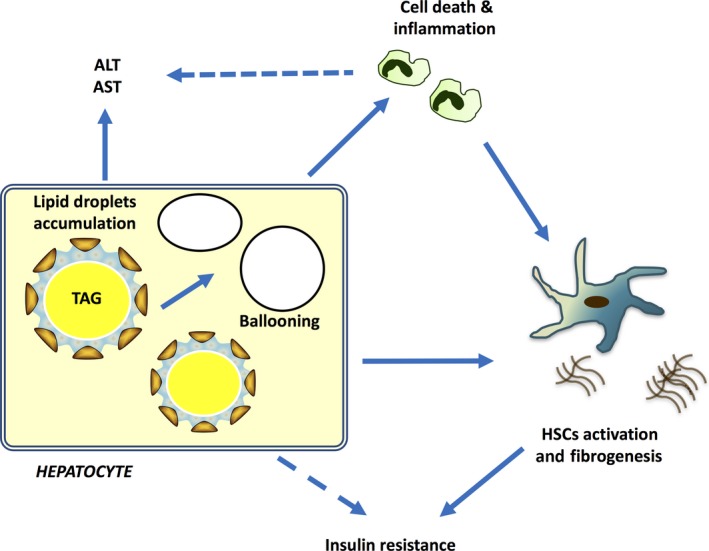
Schematic representation of main study findings. Hepatic fat accumulation is causally associated with increased liver enzymes, hepatocellular damage, necroinflammation and fibrosis. TAG, triglycerides, AST, aspartate aminotransferases, ALT, alanine aminotransferases, HSCs, hepatic stellate cells.

The current study represents the first attempt to apply a formal Mendelian randomization framework to test whether hepatic fat is causally related to liver damage and fibrosis. The main findings of this work are as follows: (i) the magnitude of association of genetic risk variants with fibrosis severity, the major prognostic determinant in NAFLD, is proportional to their effect on steatosis, suggesting that the association between these genetic variants and liver disease is explained by hepatic fat accumulation; (ii) the association between hepatic fat accumulation and fibrosis was independent of inflammation, suggesting that it is not influenced by pleiotropic effects of genetic variants on inflammation; (iii) the effect size of genetically predicted steatosis on fibrosis was consistent with that of observational steatosis, suggesting that long‐term exposure to steatosis causes fibrosis independently of confounders; (iv) the association of genetically determined hepatic fat with insulin resistance was restricted to high‐risk individuals, suggesting that insulin resistance is not a direct consequence of hepatic fat accumulation but a phenomenon possibly mediated by liver damage. A schematic representation of these study findings is presented in Fig. 4.

Although the associations of the four gene variants included in our risk score with NAFLD progression have been previously reported 17, 18, whether these effects are consistent with a causal role of hepatic fat or are explained by other pleiotropic effects of the genetic variants has not been examined so far. Here, we observed a clear dose–response relationship between the genetic effects on hepatic fat content and liver damage, in particular the severity of fibrosis, the major prognostic determinant in patients with NAFLD 33. Furthermore, genetically determined liver fat was strongly associated with liver fibrosis and cirrhosis in the UK Biobank database. These data suggest that the impact of these variants on liver damage predisposition is mainly determined by hepatic fat accumulation.

To increase statistical power, we constructed a GRS from the four risk variants. The GRS was strongly associated with hepatic fat content and with aminotransferase levels, as well as with the entire spectrum of histological liver damage. Interestingly, the estimated causal effect of hepatic steatosis was larger than the observational association of histological steatosis with fibrosis. This likely reflects the lifelong exposure to steatosis in carriers of steatogenic variants 11 and suggests that the histological grade of steatosis at the time of biopsy may underestimate the full impact of hepatic fat accumulation on fibrosis. In addition, the causal effect of hepatic fat on fibrosis was partly independent of disease activity at the time of biopsy, supporting the active role of hepatic fat accumulation in inducing progressive liver disease over the entire lifetime. These data are consistent with experimental evidence linking hepatic fat accumulation and insulin resistance with fibrosis in NAFLD, independent of inflammatory pathways 34.

Hepatic steatosis has been hypothesized to represent an ‘innocent bystander’ in the pathogenesis of NASH, or even a protective response to increased free fatty acids availability 35. In contrast to these hypotheses, rare mutations predisposing to severe hepatic fat accumulation frequently lead to cirrhosis and hepatocellular carcinoma even in the absence of other hepatotoxic insults 36. Furthermore, a recent population‐based twin study found that liver steatosis and fibrosis tend to be co‐inherited 37. The present results suggest that hepatic steatosis promotes the full spectrum of liver disease. Potential mechanisms include reduced ability to incorporate fatty acids in lipid droplets under stress conditions, spillover or peroxidation of fatty acids and activation of lipid‐droplet dependent signalling pathways 38. However, these results do not necessarily imply that triglyceride accumulation are the driver of liver disease progression, because NAFLD risk variant are associated with hepatic accumulation of different lipid species 39. Therefore, experimental studies are still required to investigate the mechanism underlying this association.

Finally, we found that hepatic steatosis is causally associated with insulin resistance in individuals at risk of NASH because of severe liver disease or obesity, in a liver damage dependent fashion. The association between genetically determined hepatic fat and insulin resistance was not observed in the DHS, possibly due to the low prevalence of individuals with advanced liver fibrosis in this general population. Alternatively, the lack of association may be a result of reduced power, owing to the fact that the impact of the genetic risk variants for NAFLD on liver damage was more marked in individuals with acquired risk factors such as obesity 40. The steatogenic variants were associated with an increased T2D risk in the largest T2D GWAS 30 and in a recently published study including >300 000 individuals with metabolic characterization 41. In our study, the association between a genetically increased liver fat content and insulin resistance appeared to depend on the presence of liver damage. This might reflect decreased hepatic insulin signalling induced by accumulation of specific lipid species or a fibrosis‐mediated decrease in hepatic insulin clearance 42, 43. Taken together, these data support the hypothesis that inhibition of hepatic fat accumulation might lead to improvement of insulin resistance and a reduction in risk of T2D, secondary to the improvement of liver disease.

There are limitations to our study. We evaluated a small number of variants, but given the high heritability of NAFLD 31, the GRS explained a larger fraction of phenotypic variability than most scores based on a large number of variants in similar studies conducted for other complex traits 32. An underlying assumption of the Mendelian randomization method is that genetic variants used as proxies for exposure influence the outcome solely via their effect on the exposure and not via pleiotropic effects. *TM6SF2* rs58542926 (E167K) impairs hepatic secretion of lipoproteins and reduces plasma levels of triglycerides and cholesterol. However, reduced levels of lipoproteins are not likely to directly cause liver damage. *GCKR* rs1260326 (P446L) increases the trapping of glucose in the liver and has an impact on plasma levels of glucose and insulin. This may pose a problem when using the variant as a proxy for hepatic fat with T2D as an outcome. *MBOAT7* rs641738 is associated with changes in circulating levels of phosphatidylinositols 17, which are unlikely to influence liver damage. Even though we cannot rule out a possibility that *PNPLA3* rs738409 (I148M) also promotes liver damage by alternate mechanisms, for example by altering retinol metabolism 44, our results held consistent when excluding the *PNPLA3* rs738409 (I148M) from the GRS. In summary, pleiotropic effects are unlikely to explain the main findings of our study, even if pleiotropy remains an unavoidable limitation of the Mendelian randomization approach 11, 45.

To reduce bias, we performed several sensitivity analyses, including the evaluation of alternative GRS, and validated the results in three independent cohorts. Steatosis grade (a categorical phenotype) was approximated to a continuous trait in the LBC. However, using steatosis grade as an ordinal categorical trait did not change the results. Finally, direct measurement of hepatic fat content was not available in the SOS, and causes of fatty liver other than NAFLD could not be ruled out in the DHS and SOS.

## Conclusion

Genetic variants that increase hepatic fat content are associated with an increase in biochemical markers of liver damage and the risk of hepatic fibrosis, to the degree predicted by their steatogenic effects. This supports the hypothesis that hepatic steatosis per se is likely to be a causal risk factor for the development of liver fibrosis, independent of inflammation. In addition, genetic variants that increase hepatic fat content were associated with modest increases in insulin resistance and risk of T2D. However, these associations were only observed in individuals with liver disease, suggesting that liver disease, rather than hepatic steatosis, could be the underlying causal factor. Taken together, these data suggest that interventions aimed at reducing hepatic steatosis are likely to have long‐term beneficial effects on liver disease and potentially on insulin resistance in patients with NAFLD.

## Conflict of interest statement

Authors declare that they do not have any conflict of interest relevant to this manuscript. SR has been consulting for Chiesi Farmaceutici Group, Amgen, Sanofi, Novonordisk, Akcea therapeutics, Genzyme, AstraZeneca and Aegerion in the last 5 years.

## Funding

This work was supported by Associazione Italiana Ricerca sul Cancro, myFIRST Grant AIRC (16888), Molecular Medicine Grant Fondazione IRCCS Ca' Granda and INGM 2014, Ricerca Corrente Fondazione Ca' Granda IRCCS Policlinico of Milan, Associazione Malattie Metaboliche del Fegato ONLUS (L.V.); the National Institutes of Health (NIH), National Institute of Diabetes and Digestive and Kidney Diseases (R01 DK090066); NIH, National Heart, Lung, and Blood Institute (P01 HL20948); NIH, National Center for Advancing Translational Sciences (UL1TR001105) (J.K.); the Swedish Research Council (Vetenskapsrådet), grants 254439006 and K2013‐54X‐11285‐19; the Swedish Heart Lung Foundation (244439007), the Swedish federal government funding under the Agreement on Medical Training and Medical Research (76290), Sahlgrenska University Hospital ALF research grant ALFGBG‐428911, the Novonordisk Foundation Grant for Excellence in Endocrinology (244439012), the Swedish Diabetes Foundation (DIA 2014‐052) (S.R.), the Wilhelm and Martina Lundgren Science Fund (R.M.M, P.P, and S.R.), the Nilsson‐Ehle funds from the Fysiografiska Sällsk‐apet in Lund (R.M.M.), and the Danish Council for Independent Research, Medical Sciences (Sapere Aude 4004‐00398) (S.S.).

## Supporting information


**Data S1.** Supplementary methods.
**Data S2.** Supplementary results.
**Table S1.** Noninvasive fibrosis scores tested in the DHS for association with hepatic fat content.
**Table S2.** Coefficient used to develop genetic risk scores (GRS) applied in the study cohorts (full models and sensitivity analysis conducted by removing one genetic variant at time).
**Table S3.** Association of genetic variants included in the GRS (*PNPLA3* rs738409 I148M, *TM6SF2* rs58542926 E167K, *GCKR* rs1260326 P446L, and *MBOAT7* rs641738) with liver damage and clinical parameters epidemiologically associated with NAFLD, in the LBC (*N* = 1515), SOS (*N* = 3329), and DHS (*N* = 4570). Estimates of β _variant‐feature_ are reported for adjusted models.
**Table S4.** Causal effect estimates of hepatic fat content, as evaluated by instrumental regression analysis by the 2SLS method, on metabolic and hepatic correlates of NAFLD in the LBC (*n* = 1515 at risk of NASH) and the DHS (*n* = 2736 from the general population).
**Table S5.** Sensitivity analyses of IV regression analysis evaluating the causal role of hepatic fat on the hepatic and metabolic NAFLD features in the study cohorts with alternative GRS excluding one genetic risk variant per time. Estimate of causality ‐ β coefficient ‐ (95% confidence intervals) and *P* values are reported for adjusted models.
**Table S6.** Associations of steatogenic variants with ICD‐code defined liver endpoints in 408 455 participants from UKBiobank.
**Table S7.** Weights used in GRS construction in DHS.
**Table S8**. Impact of the LYPLAL1 rs12137855 variant on hepatic and metabolic features of NAFLD in the DHS. Estimate of β_variant‐feature_ are reported for adjusted models.
**Table S9**. Association of genetically determined hepatic fat with liver damage and metabolic features, using alternative GRS‐S1 and GRS‐S2.
**Table S10.** Sensitivity analysis: causal effect estimates of hepatic fat content on hepatic and metabolic correlates of NAFLD in the LBC, stratified by modality of recruitment (Hepatology clinics vs. Bariatric surgery centers) in the LBC.
**Table S11.** Causal effect estimates of hepatic fat content on hepatic and metabolic correlates of NAFLD in the DHS, stratified by ethnicity.
**Figure S1**. Schematic representation of the principles of Mendelian randomization**.**
**Figure S2.** Frequency distribution of the number of risk variants for hepatic fat accumulation in *PNPLA3*,* TM6SF2*,* GCKR*, and *MBOAT7* carried by each individual in the LBC, SOS, and in the DHS.
**Figure S3**. Comparison of the impact of the evaluated common risk variants in *PNPLA3*,* TM6SF2*,* GCKR*, and *MBOAT7* on steatosis grade vs. fibrosis stage in the LBC by ordinal regression analysis.
**Figure S4**. Comparison of the impact of the evaluated common risk variants in *PNPLA3*,* TM6SF2*,* GCKR*, and *MBOAT7* on hepatic fat content vs. other liver diseases (ICD9‐K76, including NAFLD) in the UKBiobank (*n* = 408 455). Beta coefficients and 95% confidence intervals are shown.
