# Supplementary material for: Causal relationship of hepatic fat with liver damage and insulin resistance in nonalcoholic fatty liver
Source: J Intern Med. 2017 Dec 27;283(4):356–70. doi: 10.1111/joim.12719 (PMC5900872; doi:10.1111/joim.12719)

**SUPPLEMENTARY MATERIAL**

**Causal association of hepatic fat with liver damage and insulin resistance in nonalcoholic fatty liver**

Paola Dongiovanni, Stefan Stender, Alessandro Pietrelli, Rosellina M Mancina, Annalisa Cespiati, Salvatore Petta, Serena Pelusi, Piero Pingitore, Sara Badiali, Marco Maggioni, Ville Mannisto, Stefania Grimaudo, Rosaria Maria Pipitone, Jussi Pihlajamaki, Antonio Craxi, Magdalena Taube, Lena MS Carlsson, Silvia Fargion, Stefano Romeo, Julia Kozlitina, and Luca Valenti.

**SUPPLEMENTARY METHODS**

***Study cohorts***

Part of the LBC has previously been described [1, 2]. Briefly, a total of 1515 adult individuals of European descent were consecutively enrolled from the Metabolic Liver Diseases outpatient service and bariatric surgery center, Fondazione IRCCS Ca’ Granda Ospedale Policlinico Milano, Milan, Italy [3], the Gastrointestinal & Liver Unit of the Palermo University Hospital, Palermo, Italy [4], and from the Northern Savo Hospital District, Kuopio, Finland [5]. Inclusion criteria were liver biopsy for suspected NASH or severe obesity, availability of DNA samples and clinical data. Individuals with increased alcohol intake (men, >30 g/day; women, >20 g/day), viral and autoimmune hepatitis or other causes of liver disease were excluded. Compared to previous studies [3], we did not consider individuals in the developmental age, and as it is an open cohort, we recruited new individuals. The study conformed to Declaration of Helsinki and was approved by the Institutional Review Board of the Fondazione Ca’ Granda IRCCS of Milan.

The SOS study cohort has been described [6]. Briefly, the SOS in prospective controlled study evaluating severely obese individuals who underwent bariatric surgery and matched controls who received usual care in Sweden. A total of 3329 subjects who had complete baseline metabolic characterization and who were successfully genotyped were included. Hepatic fat was not directly quantified in this cohort, therefore we applied a genetic score derived from severely obese individuals included in the LBC. In the SOS, information about ethnicity is not available, as it was not consented by the Swedish law, but all participants were resident of Sweden and most of presumed European descent.

The DHS is a multiethnic population-based probability sample of Dallas County residents. The study design and recruitment procedures have been previously described in details [7]. The original cohort was enrolled between 2000 and 2002, and all participants as well as their spouses or significant others were invited for a repeat evaluation in 2007-2009 (DHS-2). The study was approved by the Institutional Review Board of University of Texas Southwestern Medical Center and all individuals provided written informed consent. Each participant completed a detailed staff-administered survey, including questions about demographics, socioeconomic status, medical history, and current medication use, and underwent a health examination that involved measurement of blood pressure, anthropometry, blood and urine sample collection, and imaging studies. Ethnicity was self-reported. Hepatic triglyceride content was measured with proton magnetic resonance spectroscopy (^1^H-MRS) as previously described[8, 9]. The present investigation includes a total of 4570 individuals (including 52% African-Americans, 29% European-Americans, 16% Hispanics, and 3% of other ethnicities), who completed a clinic visit and provided blood samples for genetic analysis. Of these, 2736 individuals had available measures of liver fat.

***Study design***

We used Mendelian randomization to estimate the causal effect of hepatic steatosis on metabolic features and liver damage associated with NAFLD. Mendelian randomization is an instrumental variable approach to infer causality in observational studies in the presence of potential confounding and reverse causation [10, 11]. The modifiable exposure in this study was hepatic fat (steatosis), as assessed by either histological evaluation or nuclear magnetic spectroscopy. Variants in four established risk loci for hepatic fat accumulation and a genetic risk score (GRS) for predisposition to steatosis composed of these variants were used as instruments (see statistical analysis) [2, 12]. Causal effect of hepatic fat on histological and metabolic features of NAFLD are estimated by examining the GRS for association with observed hepatic fat content, as well as with each NAFLD feature separately using a triangular approach (Figure S1) [13]:

1. The observational association between histological hepatic fat (steatosis) and hepatic and metabolic NAFLD features was examined in a traditional cross-sectional study design. These observational associations (association magnitude: β _HF-feature_) can arise from both directions and can be biased due to confounding.
2. GRS is confirmed to be associated with hepatic fat (β _HF-steatosis_).
3. The association between GRS and each NAFLD feature is tested (β_GRS-feature_). The genetic effect on NAFLD features is assumed to be mediated by hepatic fat. Since genetic variants are inherited randomly at conception, transmission of the effects may be assumed independent of confounders. Further, genetic variation cannot be modified by phenotype (NAFLD features), therefore ruling out reverse causation. The instrumental variable (IV; causal effect) estimate, β _IV_ is the GRS association with NAFLD feature divided by the GRS association with hepatic fat (β _GRS-feature/_ β _GRS-HF_).
4. If hepatic fat exerts a causal, non-confounded, effect on a NAFLD feature, then the causal estimate (β _IV_) is expected to be of a similar magnitude to that observed in the cross-sectional analysis (β _HF-feature_).

***Detailed statistical analysis***

For descriptive statistics, continuous variables are shown as mean and standard deviation or median and interquartile range for highly skewed biological variables. Variables with skewed distributions were logarithmically or inverse normally transformed before analyses. All genetic analyses were calculated by using an additive model.

We used four established risk variants for hepatic fat accumulation [2, 12] and a genetic risk score (GRS) composed of these variants as instruments in Mendelian randomization analysis. Genotype frequencies of the four variants were in agreement with Hardy-Weinberg proportions (p>0.1). The association of each genetic variant with hepatic steatosis (as assessed by either histological evaluation or nuclear magnetic spectroscopy) was estimated using linear regression models, in all individuals with available measures of hepatic steatosis (1515 from LBC and 2736 from DHS). Since steatosis was graded on an ordinal categorical scale in LBC, the relationship between genetic variants and steatosis grade was also estimated using ordinal regression models adjusted for clinical confounders. The results based on these models were not materially different from the results obtained based on linear regression models. Therefore, we decided to use beta coefficients from linear regression models for our primary analysis, for consistency across cohorts. Genetic risk score was calculated across all study participants by summing the number of steatosis predisposing alleles, weighted by their effect size on steatosis. For SOS, we used the effect sizes derived from the LBC participants recruited for severe obesity, since subjects had similar ancestry and clinical characteristics. The coefficients used to derive GRS are reported in Table S2. These were strongly correlated with the observed steatosis, explaining 7.0% and 3.5% of its variability in the LBC and DHS, respectively.

Observational associations of hepatic steatosis with NAFLD and metabolic features (β _HF-feature_) were assessed using linear and logistic regression models, with steatosis as the explanatory variable, and each feature as the outcome. Next, we estimated the association between GRS and each NAFLD and metabolic features (β _GRS-feature_), using linear and logistic models for continuous and binary outcomes, respectively. The causal effect of hepatic steatosis on NAFLD and metabolic features (β _IV_) was estimated using the Wald (ratio) method, with standard errors approximated by the delta method. That is, the causal effect estimate was obtained as the ratio of the GRS association with NAFLD or metabolic feature divided by the GRS association with hepatic fat (β _GRS-feature_ / β _GRS-HF_). To avoid weak instrumental variable bias, we checked that the F-statistic for association between GRS and hepatic fat was greater than 10, which indicates that the instrumental variable is unlikely to be weak. In a sensitivity analysis, we confirmed causal estimates by a two-stage least squares (2SLS) regression procedure (using the ‘ivreg’ command in the AER package in R) in the LBC and a subset of DHS participants with available measures of HTGC.

All models were adjusted for age, sex, recruitment center (in the LBC), ethnicity (in DHS), body mass index (BMI), and statin use. Additional analyses were performed with further adjustment for disease activity or fibrosis severity.

Since most of the considered risk variants (in *PNPLA3*, *TM6SF2* and *GCKR*) have likely pleiotropic effects on circulating total cholesterol and triglycerides [14], they were not valid instruments for testing the causal effect on dyslipidemia. Therefore, these outcomes were not considered. We also conducted sensitivity analysis by calculating alternative GRS by excluding one genetic variant at the time to check for the robustness of associations.

Associations with ICD-code defined liver endpoints were extracted from <http://geneatlas.roslin.ed.ac.uk/>, a publically available database of genetic associations in the UK Biobank (n=408,455). Beta-coefficients and P-values were extracted for associations with ICD-10 K76 (‘other diseases of liver’) and K74 (‘fibrosis and cirrhosis of liver’).

Statistical analyses were carried out using the JMP 12.0 (SAS Institute, Cary, NC, USA) and R statistical analysis software version 3.3.2 (http://www.R-project.org/). P-values <0.05 were considered nominally statistically significant. For results interpretation, we applied multiple testing correction for the number independent outcomes in each cohort using the Bonferroni method.

**SUPPLEMENTARY RESULTS**

***Additional sensitivity analyses***

Another variant that has been previously reported to be associated with hepatic fat content is an intronic variant at the *LYPLAL1* locus (rs12137855) [15]. The initial association was replicated in some [16], but not in all studies [17-20]. Therefore, we sought to evaluate whether this variant was associated with hepatic fat content, liver damage, and metabolic features in a subset of DHS participants, for whom the genotype data were available (N=3,300). We further constructed two alternative GRS including the *LYPLAL1* rs12137855 variant: one based on the beta coefficient for the association of *LYPLAL1* rs12137855 with hepatic fat content in DHS (GRS-S1), and another based on beta coefficients derived from a published GWAS [15] (GRS-S2). The coefficients used for the development of alternative GRS are reported in Table S7.

The association of *LYPLAL1* rs12137855 variant with hepatic and metabolic features of NAFLD in the DHS is reported in Table S8. We saw no association between *LYPLAL1* genotype, hepatic fat, liver damage, or metabolic features in the DHS.

The associations of genetically determined hepatic fat with liver damage and insulin resistance, using alternative GRS (GRS-S1 and GRS-S2), are presented in Table S9. In both analyses, genetically determined hepatic fat remained associated with aminotransferases, but it was not associated with APRI score nor with metabolic features.

To exclude the possibility that causal estimate of hepatic fat on liver damage is influenced by selection bias, we analyzed the LBC after stratification for modality of recruitment (hepatology clinic vs. bariatric surgery). The results are shown in Table S10. A clear causal association between hepatic fat and the liver damage spectrum was evident in both groups, whereas the causal relationship of steatosis with insulin resistance was stronger in patients recruited from hepatology clinics.

Finally, the causal estimates of steatosis on NAFLD features in the DHS participants stratified for ethnicity are reported in Table S11. Hepatic fat was more strongly associated with liver enzymes in Hispanics, and there was also a consistent trend for association in European-Americans. No significant association was observed in African-Americans, a group with the lowest prevalence of the *PNPLA3* variant.

**REFERENCES**

1 Dongiovanni P, Petta S, Maglio C*, et al.* Transmembrane 6 superfamily member 2 gene variant disentangles nonalcoholic steatohepatitis from cardiovascular disease. *Hepatology* 2015; **61:** 506-14.

2 Mancina RM, Dongiovanni P, Petta S*, et al.* The MBOAT7-TMC4 Variant rs641738 Increases Risk of Nonalcoholic Fatty Liver Disease in Individuals of European Descent. *Gastroenterology* 2016; **150:** 1219-30 e6.

3 Valenti L, Nobili V, Al-Serri A*, et al.* The APOC3 T-455C and C-482T promoter region polymorphisms are not associated with the severity of liver damage independently of PNPLA3 I148M genotype in patients with nonalcoholic fatty liver. *J Hepatol* 2011; **55:** 1409-14.

4 Petta S, Miele L, Bugianesi E*, et al.* Glucokinase regulatory protein gene polymorphism affects liver fibrosis in non-alcoholic Fatty liver disease. *PLoS One* 2014; **9:** e87523.

5 Simonen M, Mannisto V, Leppanen J*, et al.* Desmosterol in human nonalcoholic steatohepatitis. *Hepatology* 2013; **58:** 976-82.

6 Carlsson LM, Peltonen M, Ahlin S*, et al.* Bariatric surgery and prevention of type 2 diabetes in Swedish obese subjects. *N Engl J Med* 2012; **367:** 695-704.

7 Victor RG, Haley RW, Willett DL*, et al.* The Dallas Heart Study: a population-based probability sample for the multidisciplinary study of ethnic differences in cardiovascular health. *Am J Cardiol* 2004; **93:** 1473-80.

8 Browning JD, Szczepaniak LS, Dobbins R*, et al.* Prevalence of hepatic steatosis in an urban population in the United States: impact of ethnicity. *Hepatology* 2004; **40:** 1387-95.

9 Szczepaniak LS, Babcock EE, Schick F*, et al.* Measurement of intracellular triglyceride stores by H spectroscopy: validation in vivo. *Am J Physiol* 1999; **276:** E977-89.

10 Lawlor DA, Harbord RM, Sterne JA, Timpson N, Davey Smith G. Mendelian randomization: using genes as instruments for making causal inferences in epidemiology. *Stat Med* 2008; **27:** 1133-63.

11 Davey Smith G, Hemani G. Mendelian randomization: genetic anchors for causal inference in epidemiological studies. *Hum Mol Genet* 2014; **23:** R89-98.

12 Dongiovanni P, Valenti L. Genetics of nonalcoholic fatty liver disease. *Metabolism* 2016; **65:** 1026-37.

13 Wurtz P, Wang Q, Kangas AJ*, et al.* Metabolic signatures of adiposity in young adults: Mendelian randomization analysis and effects of weight change. *PLoS Med* 2014; **11:** e1001765.

14 Dongiovanni P, Romeo S, Valenti L. Genetic Factors in the Pathogenesis of Nonalcoholic Fatty Liver and Steatohepatitis. *Biomed Res Int* 2015; **2015:** 460190.

15 Speliotes EK, Yerges-Armstrong LM, Wu J*, et al.* Genome-wide association analysis identifies variants associated with nonalcoholic fatty liver disease that have distinct effects on metabolic traits. *PLoS Genet* 2011; **7:** e1001324.

16 Flores YN, Velazquez-Cruz R, Ramirez P*, et al.* Association between PNPLA3 (rs738409), LYPLAL1 (rs12137855), PPP1R3B (rs4240624), GCKR (rs780094), and elevated transaminase levels in overweight/obese Mexican adults. *Mol Biol Rep* 2016; **43:** 1359-69.

17 Palmer ND, Musani SK, Yerges-Armstrong LM*, et al.* Characterization of European ancestry nonalcoholic fatty liver disease-associated variants in individuals of African and Hispanic descent. *Hepatology* 2013; **58:** 966-75.

18 Hernaez R, McLean J, Lazo M*, et al.* Association between variants in or near PNPLA3, GCKR, and PPP1R3B with ultrasound-defined steatosis based on data from the third National Health and Nutrition Examination Survey. *Clin Gastroenterol Hepatol* 2013; **11:** 1183-90 e2.

19 Yuan C, Lu L, An B, Jin W, Dong Q, Xin Y, Xuan S. Association Between LYPLAL1 rs12137855 Polymorphism With Ultrasound-Defined Non-Alcoholic Fatty Liver Disease in a Chinese Han Population. *Hepat Mon* 2015; **15:** e33155.

20 Wang X, Liu Z, Wang K*, et al.* Additive Effects of the Risk Alleles of PNPLA3 and TM6SF2 on Non-alcoholic Fatty Liver Disease (NAFLD) in a Chinese Population. *Frontiers in genetics* 2016; **7:** 140.

**SUPPLEMENTARY TABLES**

**Table S1. Noninvasive fibrosis scores tested in the DHS for association with hepatic fat content.**

| **Score** | **Formula** | **Threshold > F2** |
| --- | --- | --- |
| AST/ALT ratio | AST / ALT IU/L | >1.0 |
| APRI | AST / PLTs x 100 | >1.5 |
| FIB4 | age x AST/PLTs x ✔ALT | >3.25 |
| NAFLD fibrosis score | -1.675 + 0.037 × age + 0.094 × BMI + 1.13 × IFG/diabetes + 0.99 × AST/ALT – 0.013 × PLTs – 0.66 × albumin | < -1.455 = F0-F2  > +0.675 = F3-F4 |

ALT: alanine aminotransferases, AST: aspartate aminotransferases, PLTs: platelets, APRI: AST to platelets ratio index, FIB4: Fibrosis-4 index, BMI: body mass index; NAFLD: nonalcoholic fatty liver disease, IFG: impaired fasting glucose.

**Table S2. Coefficient used to develop genetic risk scores (GRS) applied in the study cohorts (full models and sensitivity analysis conducted by removing one genetic variant at time).**

|  | **Full model** | no PNPLA3 | no TM6SF2 | no GCKR | no MBOAT7 |
| --- | --- | --- | --- | --- | --- |
| **LBC** |  |  |  |  |  |
| Constant | 0.408 | 0.465 | 0.420 | 0.436 | 0.420 |
| PNPLA3 alleles | 0.094 | - | 0.095 | 0.095 | 0.094 |
| TM6SF2 alleles | 0.108 | 0.112 | - | 0.110 | 0.108 |
| GCKR alleles | 0.027 | 0.03 | 0.030 | - | 0.027 |
| MBOAT7 alleles | 0.014 | 0.017 | 0.013 | 0.014 | ­­- |
| F- statistic | 29 | 13 | 29 | 36 | 38 |
| P value | <10^-16^ | 1.2*10^-8^ | <10^-16^ | <10^-16^ | <10^-16^ |
| Variance explained | 7.0% | 2.6% | 5.3% | 6.7% | 6.9% |
| **LBC obese*** |  |  |  |  |  |
| Constant | 0.346 | 0.389 | 0.357 | 0.369 | 0.362 |
| PNPLA3 alleles | 0.086 | - | 0.084 | 0.087 | 0.087 |
| TM6SF2 alleles | 0.130 | 0.123 | - | 0.132 | 0.131 |
| GCKR alleles | 0.025 | 0.028 | 0.028 | - | 0.025 |
| MBOAT7 alleles | 0.019 | 0.021 | 0.021 | 0.019 | - |
| F- statistic | 10 | 4 | 7 | 10 | 10 |
| P value | 4.5*10^-6^ | 0.0059 | 0.0001 | 2.9*10^-6^ | 2.1*10^-6^ |
| Variance explained | 4.7% | 2.0% | 2.7% | 4.4% | 4.5% |
| **DHS** |  |  |  |  |  |
| PNPLA3 alleles | 0.266 | na | 0.266 | 0.266 | 0.264 |
| TM6SF2 alleles | 0.274 | 0.275 | Na | 0.271 | 0.273 |
| GCKR alleles | 0.065 | 0.068 | 0.061 | Na | 0.065 |
| MBOAT7 alleles | 0.063 | 0.059 | 0.063 | 0.063 | na |
| F- statistic | 99 | 30 | 78 | 94 | 94 |
| P value | <2.2*10^-16^ | 5.8*10^-8^ | <2.2*10^-16^ | <2.2*10^-16^ | <2.2*10^-16^ |
| Variance explained | 3.5% | 1.1% | 2.8% | 3.3% | 3.3% |

LBC: Liver Biopsy Cohort, SOS: Swedish Obese Subjects Study, DHS: Dallas Heart Study; NA: not addressed. *Used for derivation of the GRS in SOS.

**Table S3. Association of genetic variants included in the GRS (*PNPLA3* rs738409 I148M, *TM6SF2* rs58542926 E167K, *GCKR* rs1260326 P446L, and *MBOAT7* rs641738) with liver damage and clinical parameters epidemiologically associated with NAFLD, in the LBC (N=1515), SOS (N=3329), and DHS (N=4570). Estimates of β _variant-feature_ are reported for adjusted models.**

|  |  |  | **LBC** |  |  | **SOS** |  | | **DHS** |  |  |
| --- | --- | --- | --- | --- | --- | --- | --- | --- | --- | --- | --- |
| **Outcome** | **Variant** | **Beta** | **95% c.i.** | **p value** | **Beta** | **95% c.i.** | **p value** | **Beta** | **95% c.i.** | **p value** | |
| ALT | PNPLA3 | +0.45 | (0.33-0.58) | 9*10^-13^ | +0.13 | (0.08-0.14) | 9.5*10^-13^ | +0.10 | (0.05-0.15) | 1.9*10^-5^ | |
|  | TM6SF2 | +0.15 | (-0.09-0.38) | 0.22 | +0.07 | (0.04-0.12) | 0.00018 | +0.09 | (0.01-0.18) | 0.035 | |
|  | GCKR | +0.12 | (0-0.24) | 0.060 | 0 | (-0.02-0.03) | 0.80 | +0.01 | (-0.03-0.06) | 0.64 | |
|  | MBOAT7 | +0.01 | (-0.11-0.13) | 0.87 | +0.02 | (-0.01-0.04) | 0.24 | +0.00 | (-0.04-0.04) | 0.97 | |
| AST | PNPLA3 | +0.45 | (0.32-0.58) | 1.6*10^-11^ | +0.13 | (0.06-0.11) | 2.1*10^-14^ | +0.08 | (0.03-0.13) | 0.0015 | |
|  | TM6SF2 | +0.06 | (-0.18-0.31) | 0.61 | +0.06 | (0.02-0.08) | 0.001 | +0.04 | (-0.05-0.13) | 0.43 | |
|  | GCKR | +0.13 | (0-0.26) | 0.048 | +0.02 | (-0.01-0.0.4) | 0.24 | +0.06 | (0.02-0.11) | 0.0082 | |
|  | MBOAT7 | 0 | (-0.12-0.13) | 0.96 | +0.01 | (-0.01-0.03) | 0.52 | -0.02 | (-0.07-0.02) | 0.24 | |
| STEATOSIS | PNPLA3 | +0.62 | (0.48-0.76) | 5*10^-18^ |  | - |  | +0.27 | (0.21-0.33) | 2.0*10^-20^ | |
|  | TM6SF2 | +0.69 | (0.43-0.95) | 3.5*10^-7^ |  | - |  | +0.26 | (0.15-0.37) | 3.3*10^-6^ | |
|  | GCKR | +0.20 | (0.06-0.34) | 0.005 |  | - |  | +0.09 | (0.04-0.15) | 7.8*10^-4^ | |
|  | MBOAT7 | +0.12 | (-0.02-0.25) | 0.11 |  | - |  | +0.05 | (0.01-0.1) | 0.026 | |
| Necroinflammation | PNPLA3 | +0.41 | (0.26-0.55) | 1.7*10^-8^ |  | - |  |  | - |  | |
|  | TM6SF2 | +0.32 | (0.05-0.58) | 0.018 |  | - |  |  | - |  | |
|  | GCKR | +0.25 | (0.11-0.39) | 5.1*10^-4^ |  | - |  |  | - |  | |
|  | MBOAT7 | +0.20 | (0.06-0.37) | 0.0047 |  | - |  |  | - |  | |
| Ballooning | PNPLA3 | +0.28 | (0.15-0.42) | 3*10^-5^ |  | - |  |  | - |  | |
|  | TM6SF2 | +0.27 | (0.02-0.52) | 0.032 |  | - |  |  | - |  | |
|  | GCKR | +0.21 | (0.08-0.35) | 0.0016 |  | - |  |  | - |  | |
|  | MBOAT7 | +0.02 | (-0.10-0.15) | 0.54 |  | - |  |  | - |  | |
| Fibrosis | PNPLA3 | +0.46 | (0.32-0.60) | 5*10^-11^ |  | - |  |  | - |  | |
|  | TM6SF2 | +0.43 | (0.17-0.69) | 0.0012 |  | - |  |  | - |  | |
|  | GCKR | +0.18 | (0.04-0.32­) | 0.010 |  | - |  |  | - |  | |
|  | MBOAT7 | +0.12 | (0-0.26) | 0.048 |  | - |  |  | - |  | |
| T2D | PNPLA3 | -0.06 | (-0.20- 0.08) | 0.38 | +0.11 | (-0.05-0.30) | 0.22 | +0.05 | (-0.12-0.22) | 0.58 | |
|  | TM6SF2 | +0.07 | (-0.19-0.32) | 0.60 | +0.11 | (-0.1-0.4) | 0.49 | -0.24 | (-0.59-0.11) | 0.19 | |
|  | GCKR | -0.08 | (-0.21-0.06) | 0.26 | +0.07 | (-0.07-0.24) | 0.34 | -0.10 | (-0.28-0.07) | 0.24 | |
|  | MBOAT7 | -0.06 | (-0.19-0.07) | 0.237 | +0.10 | (-0.06-0.26) | 0.19 | +0.03 | (-0.12-0.17) | 0.72 | |
| Hypertension | PNPLA3 | 0.02 | (-0.13-0.17) | 0.77 | -0.07 | (-0.17-0.06) | 0.27 | -0.14 | (-0.27- -0.01) | 0.04 | |
|  | TM6SF2 | -0.04 | (-0.31-0.23) | 0.80 | +0.11 | (-0.08-0.13) | 0.28 | -0.04 | (-0.29-0.22) | 0.77 | |
|  | GCKR | +0.10 | (-0.05-0.25) | 0.18 | +0.12 | (0.04-0.13) | 0.008 | -0.03 | (-0.16-0.10) | 0.65 | |
|  | MBOAT7 | -0.08 | (-0.23-0.06) | 0.26 | +0.02 | (-0.09-0.11) | 0.78 | -0.07 | (-0.18-0.04) | 0.21 | |
| HOMA-IR | PNPLA3 | +0.17 | (0.02-0.32) | 0.030 | +0.04 | (0.01-0.09) | 0.007 | +0.03 | (-0.02-0.07) | 0.26 | |
|  | TM6SF2 | +0.25 | (-0.03-0.54) | 0.076 | +0.01 | (-0.04-0.09) | 0.20 | +0.03 | (-0.06-0.11) | 0.52 | |
|  | GCKR | -0.04 | (-0.19-0.10) | 0.55 | -0.01 | (-0.04--0.02) | 0.52 | -0.06 | (-0.10- -0.01) | 0.013 | |
|  | MBOAT7 | +0.08 | (-0.07-0.23) | 0.28 | +0.02 | (-0.01-0.05) | 0.18 | +0.02 | (-0.01-0.06) | 0.2 | |
| Total cholesterol | PNPLA3 | +0.08 | (-0.06-0.22) | 0.27 | -0.04 | (-0.13- -0.02) | 0.033 | +0.00 | (-0.05-0.05) | 0.9 | |
|  | TM6SF2 | -0.40 | (0.66--0.14) | 0.0028 | -0.08 | (-0.29- -0.11) | 8.4*10^-6^ | -0.16 | (-0.25- -0.07) | 9.1*10^-4^ | |
|  | GCKR | +0.19 | (0.05-0.32) | 0.009 | +0.06 | (0.04-0.15) | 0.0003 | +0.10 | (0.05-0.15) | 1.3*10^-4^ | |
|  | MBOAT7 | +0.03 | (-0.11-0.16) | 0.71 | 0 | (-0.05-0.05) | 0.95 | -0.01 | (-0.05-0.03) | 0.58 | |
| HDL | PNPLA3 | -0.12 | (-0.26-0.01) | 0.084 | -0.01 | (-0.03--0.01) | 0.41 | -0.05 | (-0.09-0.00) | 0.049 | |
|  | TM6SF2 | -0.03 | (-0.29-0.23) | 0.82 | -0.02 | (-0.04-0.01) | 0.16 | +0.00 | (-0.09-0.09) | 0.98 | |
|  | GCKR | -0.01 | (-0.15-0.13) | 0.88 | -0.02 | (-0.03-0) | 0.17 | -0.01 | (-0.05-0.04) | 0.73 | |
|  | MBOAT7 | -0.07 | (-0.20-0.06) | 0.33 | -0.02 | (-0.03-0) | 0.15 | -0.01 | (-0.05-0.03) | 0.66 | |
| Triglycerides | PNPLA3 | +0.16 | (0.02-0.31) | 0.024 | -0.04 | (-0.06-0) | 0.040 | +0.01 | (-0.03-0.06) | 0.56 | |
|  | TM6SF2 | -0.50 | (-0.76- -0.23) | 0.0002 | -0.07 | (-0.12- -0.04) | 2.3*10^-5^ | -0.10 | (-0.19- -0.01) | 0.031 | |
|  | GCKR | +0.33 | (0.19-0.47) | 4.8*10^-6^ | +0.12 | (0.07-0.12) | 1.8*10^-13^ | +0.13 | (0.08-0.17) | 1.2*10^-7^ | |
|  | MBOAT7 | +0.03 | (-0.11-0.16) | 0.67 | -0.02 | (-0.04-0.01) | 0.16 | +0.00 | (-0.04-0.04) | 0.95 | |

Adjusted standardized beta coefficients and (95% c.i.) are reported. Coefficients were adjusted for age, sex, BMI, use of statins, recruitment center in the LBC or ethnicity in the DHS. LBC: Liver Biopsy Cohort, SOS: Swedish Obese Subjects Study, DHS: Dallas Heart Study. Results were adjusted for age, sex, BMI, statin use, recruitment criterion or ethnicity when indicated in the LBC and DHS. There was no significant association between genetic risk variants analyzed, and the demographic and anthropometric parameters considered in the three cohorts. After correction for multiple comparisons, p<0.0065, p<0.008, and p<0.0071 are considered statistically significant in the LBC, SOS, and DHS, respectively.

**Table S4.** **Causal effect estimates of hepatic fat content, as evaluated by instrumental regression analysis by the 2SLS method, on metabolic and hepatic correlates of NAFLD in the LBC (n= 1515 at risk of NASH) and the DHS (n=2736 from the general population).**

| **LBC (n=1515)** | | | | |
| --- | --- | --- | --- | --- |
| **Outcome** | **Beta** | **95% c.i.** | **p value** | **WH** |
| ALT | +0.58 | (0.42-0.74) | 7.4*10^-13^ | 0.0017 |
| AST | +0.54 | (0.37-0.72) | 3.1*10^-10^ | 0.0017 |
| Necroinflammation | +0.67 | (0.50-0.83) | 8.3*10^-17^ | 0.094 |
| Ballooning | +0.49 | (0.31-0.65) | 1.0*10^-7^ | 0.10 |
| Fibrosis | +0.73 | (0.54-0.92) | 6.3*10^-13^ | 2.7*10^-5^ |
| Hypertension | +0.03 | (-0.19-0.25) | 0.78 | - |
| T2D | -0.08 | (-0.27-0.11) | 0.43 | - |
| HOMA-IR | +0.28 | (0.08-0.47) | 0.0045 | 0.76 |
| HDL | -0.15 | (-0.33-0.03) | 0.096 | 0.30 |
| **DHS (n=2736)** | | | | |
| **Outcome** | **Beta** | **95% c.i.** | **p value** | **WH** |
| ALT, IU/l | +0.34 | (0.17-0.51) | 7.6*10^-5^ | 0.38 |
| AST, IU/l | +0.32 | (0.14-0.51) | 6.8*10^-4^ | 0.042 |
| APRI score | +0.23 | (0.005-0.45) | 0.045 | 0.139 |
| HOMA-IR | -0.01 | (-0.22-0.20) | 0.92 | - |
| HDL, mmol/l | -0.16 | (-0.34-0.03) | 0.095 | - |

Adjusted standardized beta coefficients and (95% c.i.) are reported. Coefficients were adjusted for age, sex, BMI, use of statins, recruitment center in the LBC or ethnicity in the DHS. WH: Wu-Hausman p value. ALT: alanine aminotransferases; AST: aspartate aminotransferases, T2D: type 2 diabetes, HOMA-IR: homeostasis model assessment-insulin resistance index, HDL: high-density lipoprotein cholesterol, APRI: AST to platelets ratio index. After correction for multiple comparisons, p<0.0065, p<0.008, and p<0.0071 are considered statistically significant in the LBC, SOS, and DHS, respectively.

**Table S5. Sensitivity analyses of IV regression analysis evaluating the causal role of hepatic fat on the hepatic and metabolic NAFLD features in the study cohorts with alternative GRS excluding one genetic risk variant per time. Estimate of causality - β coefficient - (95% confidence intervals) and p values are reported for adjusted models.**

|  |  |  | | **LBC** |  |  | **SOS** |  |  | **DHS** |  |
| --- | --- | --- | --- | --- | --- | --- | --- | --- | --- | --- | --- |
| **Instrument** | **Outcome** | **Beta** | **95% c.i.** | | **p value** | **Beta** | **95% c.i.** | **p value** | **Beta** | **95% c.i.** | **p value** |
|  | ALT | +0.15 | (0-0.33) | | 0.053 | +0.31 | (0.16-0.45) | 3.9*10^-5^ | +0.29 | (-0.04-0.62) | 0.089 |
|  | AST | +0.11 | (-0.04-0.31) | | 0.18 | +0.28 | (0.13-0.43) | 2.7*10^-4^ | +0.28 | (-0.08-0.65) | 0.13 |
| **GRS - no PNPLA3** | T2D | -0.04 | (-0.33-0.18) | | 0.77 | +0.12 | (-0.03-0.28) | 0.12 | -0.79 | (-2.22-0.64) | 0.27 |
|  | HTN | 0 | (-0.47-0.53) | | 0.91 | +0.15 | (-0.002-0.30) | 0.05 | -0.01 | (-0.91-0.9) | 0.99 |
|  | HOMA-IR | +0.15 | (-0.04-0.38) | | 0.13 | +0.10 | (-0.05-0.24) | 0.20 | +0.03 | (-0.35-0.41) | 0.86 |
|  | HDL | -0.04 | (-0.22-0.15) | | 0.63 | -0.16 | (-0.32- -0.01) | 0.03 | +0.03 | (-0.32-0.39) | 0.85 |
|  | NecroInf | +0.42 | (0.22-0.60) | | 2*10^-5^ | - | - | - | - | - | - |
|  | Ballooning | +0.30 | (0.11-0.48) | | 0.00087 | - | - | - | - | - | - |
|  | Fibrosis | +0.42 | (0.22-0.60) | | 1*10^-5^ | - | - | - | - | - | - |
|  | ALT | +0.60 | (0.46-0.81) | | 5*10^-13^ | +0.56 | (0.41-0.70) | 7*10^-14^ | +0.33 | (0.13-0.53) | 0.0011 |
|  | AST | +0.60 | (0.46-0.81) | | 5*10^-12^ | +0.56 | (0.41-0.71) | 2*10^-13^ | +0.36 | (0.13-0.58) | 0.0018 |
| **GRS – no TM6SF2** | T2D | -0.11 | (-0.29-0.04) | | 0.21 | +0.14 | (-0.01-0.30) | 0.07 | -0.49 | (-1.3-0.31) | 0.23 |
|  | HTN | +0.04 | (-0.15-0.27) | | 0.61 | +0.004 | (-0.15-0.16) | 0.96 | -0.42 | (-1-0.16) | 0.15 |
|  | HOMA-IR | +0.05 | (0.19-0.42) | | 0.047 | +0.19 | (0.05-0.34) | 0.01 | -0.06 | (-0.29-0.18) | 0.63 |
|  | HDL | -0.15 | (-0.33-0) | | 0.076 | -0.12 | (-0.27-0.03) | 0.11 | -0.22 | (-0.43--0.01) | 0.04 |
|  | NecroInf | +0.63 | (0.45-0.84) | | 2*10^-11^ | - | - | - | - | - | - |
|  | Ballooning | +0.45 | (0.27-0.63) | | 1*10^-6^ | - | - | - | - | - | - |
|  | Fibrosis | +0.66 | (0.48-0.87) | | 1*10^-12^ | - | - | - | - | - | - |
|  | ALT | +0.55 | (0.38-0.70) | | 4*10^-11^ | +0.66 | (0.51-0.80) | 8*10^-19^ | +0.35 | (0.16-0.53) | 2.0*10^-4^ |
|  | AST | +0.53 | (0.33-0.67) | | 3*10^-9^ | +0.58 | (0.43- 0.73) | 4*10^-14^ | +0.29 | (0.09-0.49) | 0.0048 |
| **GRS – no GCKR** | T2D | -0.04 | (-0.22-0.11) | | 0.57 | +0.14 | (-0.02-0.29) | 0.09 | -0.4 | (-1.14-0.34) | 0.28 |
|  | HTN | 0 | (-0.18-0.18) | | 0.97 | +0.01 | (-0.14-0.16) | 0.87 | -0.28 | (-0.81-0.24) | 0.28 |
|  | HOMA-IR | +0.30 | (0.07-0.48) | | 0.0041 | +0.22 | (0.07-0.37) | 0.003 | +0.04 | (-0.17-0.24) | 0.72 |
|  | HDL | -0.15 | (-0.33-0) | | 0.094 | -0.14 | (-0.29-0.01) | 0.07 | -0.14 | (-0.33-0.05) | 0.16 |
|  | NecroInf | +0.60 | (0.45-0.84) | | 3*10^-10^ | - | - | - | - | - | - |
|  | Ballooning | +0.42 | (0.22-0.60) | | 4*10^-6^ | - | - | - | - | - | - |
|  | Fibrosis | +0.67 | (0.54-0.87) | | 1*10^-13^ | - | - | - | - | - | - |
|  | ALT | +0.57 | (0.42-0.77) | | 8*10^-12^ | +0.63 | (0.49-0.78) | 2*10^-17^ | +0.35 | (0.17-0.54) | 1.6*10^-4^ |
|  | AST | +0.57 | (0.38-0.70) | | 5*10^-10^ | +0.59 | (0.44-0.74) | 1*10^-14^ | +0.35 | (0.14-0.55) | 9.0*10^-4^ |
| **GRS – no MBOAT7** | T2D | -0.07 | (-0.27-0.11) | | 0.48 | +0.14 | (-0.02-0.29) | 0.09 | -0.52 | (-1.27-0.23) | 0.17 |
|  | HTN | +0.03 | (-0.15-0.27) | | 0.61 | +0.05 | (-0.10-0.20) | 0.52 | -0.28 | (-0.8-0.24) | 0.29 |
|  | HOMA-IR | +0.27 | (0.03-0.46) | | 0.012 | +0.19 | (0.04-0.34) | 0.01 | -0.04 | (-0.25-0.17) | 0.71 |
|  | HDL | -0.15 | (-0.33-0.38) | | 0.12 | -0.14 | (-0.29-0.01) | 0.06 | -0.19 | (-0.38-0) | 0.055 |
|  | NecroInf | +0.63 | (0.52-0.86) | | 5*10^-11^ | - | - | - | - | - | - |
|  | Ballooning | +0.48 | (0.30-0.63) | | 2*10^-7^ | - | - | - | - | - | - |
|  | Fibrosis | +0.70 | (0.54-0.88) | | 4*10^-14^ | - | - | - | - | - | - |

Adjusted standardized beta coefficients and (95% c.i.) are reported. Coefficients were adjusted for age, sex, BMI, use of statins, recruitment center in the LBC or ethnicity in the DHS. LBC: Liver Biopsy Cohort, SOS: Swedish Obese Subjects Study, DHS: Dallas Heart Study, ALT: alanine aminotransferases; AST: aspartate aminotransferases, T2D: type 2 diabetes, HTN: arterial hypertension, HOMA-IR: homeostasis model assessment insulin resistance index, NecroInf: necroinflammation. Analysis were adjusted for age, sex, BMI, statin use, recruitment criterion or ethnicity in the LBC and DHS, respectively.

**Table S6. Associations of steatogenic variants with ICD-code defined liver endpoints in 408,455 participants from UKBiobank.**

| **Endpoint** | **Gene** | **Rs number** | **Beta** | **SE** | **p** |
| --- | --- | --- | --- | --- | --- |
| K76 other diseases of liver | *PNPLA3* | rs738409 | 0.00198 | 2.7*10^-4^ | 2.1*10^-12^ |
| (n=3351) | *TM6SF2* | rs58542926 | 0.00219 | 5.4*10^-4^ | 6.2*10^-5^ |
|  | *GCKR* | rs1260326 | 0.00041 | 2.0*10^-4^ | 0.0047 |
|  | *MBOAT7* | rs641738 | 0.00042 | 2.0*10^-4^ | 0.0075 |
|  | *LYPLAL1* | rs12137855 | -8.16*10^-5^ | 0.00025 | 0.74 |
|  |  |  |  |  |  |
| K74 fibrosis and cirrhosis of liver | *PNPLA3* | rs738409 | 0.00075 | 1.6*10^-4^ | 3.5*10^-6^ |
| (n=805) | *TM6SF2* | rs58542926 | 0.00069 | 1.8*10^-4^ | 1.7*10^-4^ |
|  | *GCKR* | rs1260326 | 0.00022 | 1.0*10^-4^ | 1.2*10^-1^ |
|  | *MBOAT7* | rs641738 | 0.00022 | 2.0*10^-4^ | 2.3*10^-2^ |
|  | *LYPLAL1* | rs12137855 | -1.50*10^-5^ | 0.00012 | 0.90 |

Data were extracted from <http://geneatlas.roslin.ed.ac.uk/> (accessed 6^th^ November, 2017), a publically available database of genetic associations in the UKBiobank. Beta: per-allele beta-coefficient estimated by a mixed linear model. The modeled effect-allele is the steatogenic allele for each single nucleotide polymorphism. SE: standard error for the beta coefficients. * *LYPLAL1* rs12137855 was not directly genotyped in UK Biobank, but was imputed (imputation quality score = 0.99427).

| **Table S7. Weights used in GRS construction in DHS.** | | | | |
| --- | --- | --- | --- | --- |
|  |  | GRS | GRS-S1 | GRS-S2 |
| Locus | Variant | Beta (DHS) | Beta (DHS) | Beta  (Speliotes et al.) |
| PNPLA3 | rs738409 | 0.2657 | 0.2653 | 0.23 |
| TM6SF2 | rs58542926 | 0.2738 | 0.2711 | 0.24 |
| GCKR | rs1260326 | 0.0647 | 0.0649 | 0.06 |
| MBOAT7 | rs641738 | 0.0629 | 0.0575 | - |
| LYPLAL1 | rs12137855(C) | - | -0.0167 | 0.08 |
| GRS - GRS used in the primary analysis; GRS-S1 - GRS including LYPLAL1 variant, based on beta coefficients in DHS; GRS-S2 - GRS including LYPLAL1 variant and excluding MBOAT7, based on beta coefficients from Speliotes *et al*. [15]. | | | | |

**Table S8. Impact of the LYPLAL1 rs12137855 variant on hepatic and metabolic features of NAFLD in the DHS. Estimate of β_variant-feature_ are reported for adjusted models.**

| **Outcome** | **N** | **Beta** | **95% c.i.** | **p-value** |
| --- | --- | --- | --- | --- |
| ALT | 3304 | -0.012 | (-0.068 - 0.044) | 0.68 |
| AST | 3304 | -0.040 | (-0.101 - 0.021) | 0.20 |
| HTGC % | 2236 | -0.014 | (-0.075 - 0.048) | 0.66 |
| T2D | 3304 | -0.101 | (-0.315 - 0.112) | 0.35 |
| Hypertension | 2861 | -0.011 | (-0.182 - 0.160) | 0.90 |
| HOMA-IR | 2782 | -0.035 | (-0.095 - 0.024) | 0.25 |
| TC | 3303 | -0.039 | (-0.103 - 0.024) | 0.23 |
| HDL | 3303 | 0.013 | (-0.046 - 0.073) | 0.66 |
| Triglycerides | 3303 | -0.031 | (-0.092 - 0.030) | 0.32 |
| APRI score | 2006 | -0.034 | (-0.109 - 0.041) | 0.37 |
|  |  |  |  |  |

ALT: alanine aminotransferases, AST: aspartate aminotransferases, HTGC %: hepatic triglyceride content, T2D: type 2 diabetes; HOMA-IR: homeostasis model assessment insulin resistance index, TC: total cholesterol, HDL: HDL cholesterol, APRI: AST to platelets ratio index.

| **Table S9**. **Association of genetically determined hepatic fat with liver damage and metabolic features, using alternative GRS-S1 and GRS-S2.** | | | | |
| --- | --- | --- | --- | --- |
| **GRS-S1** |  |  |  |  |
| **Outcome** | **N** | **Beta** | **95% c.i.** | **p-value** |
| ALT | 3299 | 0.2914 | (0.114-0.469) | 0.0013 |
| AST | 3299 | 0.2800 | (0.092-0.468) | 0.0036 |
| APRI score | 2006 | 0.1576 | (-0.066-0.381) | 0.17 |
| T2D | 3299 | 0.1421 | (-0.514-0.798) | 0.67 |
| Hypertension | 2857 | -0.1293 | (-0.657-0.399) | 0.63 |
| HOMA-IR | 2778 | 0.0867 | (-0.086-0.26) | 0.33 |
| HDL | 3298 | -0.1190 | (-0.294-0.056) | 0.18 |
| **GRS-S2** |  |  |  |  |
| **Outcome** | **N** | **Beta** | **95% c.i.** | **p-value** |
| ALT | 3299 | 0.2987 | (0.107-0.49) | 0.0022 |
| AST | 3299 | 0.2785 | (0.076-0.481) | 0.0070 |
| APRI score | 2006 | 0.1924 | (-0.049-0.434) | 0.12 |
| T2D | 3299 | -0.0473 | (-0.755-0.66) | 0.90 |
| Hypertension | 2857 | -0.1193 | (-0.685-0.447) | 0.68 |
| HOMA-IR | 2778 | 0.0329 | (-0.152-0.218) | 0.73 |
| HDL | 3298 | -0.1084 | (-0.296-0.079) | 0.26 |

ALT: alanine aminotransferases, AST: aspartate aminotransferases APRI: AST to platelets ratio index, T2D: type 2 diabetes, HOMA-IR: homeostasis model assessment insulin resistance index, HDL: HDL cholesterol.

**Table S10. Sensitivity analysis: causal effect estimates of hepatic fat content on hepatic and metabolic correlates of NAFLD in the LBC, stratified by modality of recruitment (Hepatology clinics vs. Bariatric surgery centers) in the LBC.**

|  | **HEPATOLOGY CLINICS (n=894)** | | |  |
| --- | --- | --- | --- | --- |
| **­** | **Adjusted for age, sex, center, BMI, statins** | | |  |
| **Outcome** | **Beta** | **95% c.i.** | **p value** |  |
| ALT | +0.48 | (0.22-0.70) | 6.6*10^-5^ |  |
| AST | +0.33 | (0.11-0.57) | 0.0057 |  |
| Necroinflammation | +0.48 | (0.27-0.70) | 3.4*10^-5^ |  |
| Ballooning | +0.27 | (0.07-0.48) | 0.013 |  |
| Fibrosis | +0.52 | (0.30-0.77) | 1.1*10^-5^ |  |
| Hypertension | 0 | (-0.27-0.22) | 0.86 |  |
| T2D | -0.27 | (-0.49-0) | 0.057 |  |
| HOMA-IR | +0.27 | (0-0.48) | 0.046 |  |
| HDL | -0.18 | (-0.42-0.07) | 0.14 |  |
|  | **BARIATRIC SURGERY CENTERS (n=631)** | | |  |
| **­** | **Adjusted for age, sex, BMI, statins** | | |  |
| **Outcome** | **Beta** | **95% c.i.** | **p value** |  |
| ALT | +0.38 | (0.11-0.63) | 0.0052 |  |
| AST | +0.60 | (0.30-0.87) | 3.3*10^-5^ |  |
| Necroinflammation | +0.38 | (0.07-0.63) | 0.013 |  |
| Ballooning | +0.19 | (-0.12-0.49) | 0.21 |  |
| Fibrosis | +0.53 | (0.23-0.82) | 0.00037 |  |
| Hypertension | 0 | (-0.31-0.34) | 0.90 |  |
| T2D | +0.03 | (-0.22-0.30) | 0.70 |  |
| HOMA-IR | +0.18 | (-0.17-0.67) | 0.33 |  |
| HDL | -0.17 | (-0.45-0.09) | 0.17 |  |

Adjusted standardized beta coefficients and (95% c.i.) are reported. Coefficients were adjusted for age, sex, BMI, use of statins, recruitment center in the LBC or ethnicity in the DHS. BMI: body mass index, ALT: alanine aminotransferases, AST: aspartate aminotransferases, T2D: type 2 diabetes, HOMA-IR: homeostasis model assessment-insulin resistance index, HDL: high-density lipoprotein. After correction for multiple comparisons, p<0.0065, are considered statistically significant.

**Table S11.** Causal effect estimates of hepatic fat content on hepatic and metabolic correlates of NAFLD in the DHS, stratified by ethnicity.

| **Ethnicity** | **Outcome** | **N** | **Beta** | **95% c.i.** | **p value** |
| --- | --- | --- | --- | --- | --- |
|  |  |  |  |  |  |
|  | ALT | 2297 | 0.17 | (-0.068-0.4) | 0.165 |
|  | AST | 2297 | 0.10 | (-0.145-0.338) | 0.434 |
|  | APRI | 1579 | 0.03 | (-0.249-0.312) | 0.825 |
| **AA** | T2D | 2310 | 0.11 | (-0.666-0.885) | 0.782 |
|  | HTN | 2041 | -0.72 | (-1.374--0.066) | 0.031 |
|  | HOMA-IR | 1987 | 0.24 | (0.001-0.482) | 0.049 |
|  | HDL | 2309 | -0.16 | (-0.411-0.081) | 0.190 |
|  |  |  |  |  |  |
|  | ALT | 1312 | 0.17 | (-0.071-0.413) | 0.165 |
|  | AST | 1312 | 0.19 | (-0.075-0.449) | 0.161 |
|  | APRI | 1021 | 0.17 | (-0.112-0.457) | 0.235 |
| **EA** | T2D | 1315 | -1.02 | (-2.192-0.152) | 0.088 |
|  | HTN | 1232 | -0.16 | (-0.869-0.542) | 0.650 |
|  | HOMA-IR | 1194 | -0.21 | (-0.44-0.029) | 0.086 |
|  | HDL | 1314 | -0.03 | (-0.265-0.199) | 0.783 |
|  |  |  |  |  |  |
|  | ALT | 728 | 0.86 | (0.514-1.198) | 9.2*10^-7^ |
|  | AST | 728 | 0.69 | (0.344-1.041) | 9.7*10^-5^ |
|  | APRI | 447 | 0.57 | (0.15-0.985) | 0.0077 |
| **HA** | T2D | 730 | 0.47 | (-0.655-1.593) | 0.413 |
|  | HTN | 649 | -0.90 | (-1.959-0.162) | 0.097 |
|  | HOMA-IR | 636 | 0.14 | (-0.157-0.443) | 0.350 |
|  | HDL | 729 | -0.20 | (-0.519-0.117) | 0.215 |

AA: African-Americans, EA: European-Americans, HA: Hispanic-Americans. ALT: alanine aminotransferases, AST: aspartate aminotransferases, T2D: type 2 diabetes, HTN: hypertension, HOMA-IR: homeostasis model assessment insulin resistance index, HDL: HDL cholesterol. After correction for multiple comparisons, p<0.0042 is considered statistically significant.

**SUPPLEMENTARY FIGURES**

**Figure S1**. **Schematic representation of the principles of Mendelian randomization. GRS: genetic risk score; NAFLD: nonalcoholic fatty liver disease; β coefficient of association; HF: hepatic fat; β IV: coefficient of association of the instrumental variable: a causal estimate of the impact of hepatic steatosis on NAFLD features.**

**Figure S2. Frequency distribution of the number of risk variants for hepatic fat accumulation in *PNPLA3*, *TM6SF2*, *GCKR*, and *MBOAT7* carried by each individual in the LBC, SOS, and in the DHS.** LBC vs. DHS: p=0.073; LBC vs. SOS p=0.18; DHS vs. SOS = 0.47 at Cochran-Armitage test for trend.

**Figure S3**. **Comparison of the impact of the evaluated common risk variants in *PNPLA3*, *TM6SF2*, *GCKR*, and *MBOAT7* on steatosis grade vs. fibrosis stage in the LBC by ordinal regression analysis.**

**Figure S4**. **Comparison of the impact of the evaluated common risk variants in *PNPLA3*, *TM6SF2*, *GCKR*, and *MBOAT7* on hepatic fat content vs. other liver diseases (ICD9-K76, including NAFLD) in the UKBiobank (n=408,455). Beta coefficients and 95% confidence intervals are shown.**


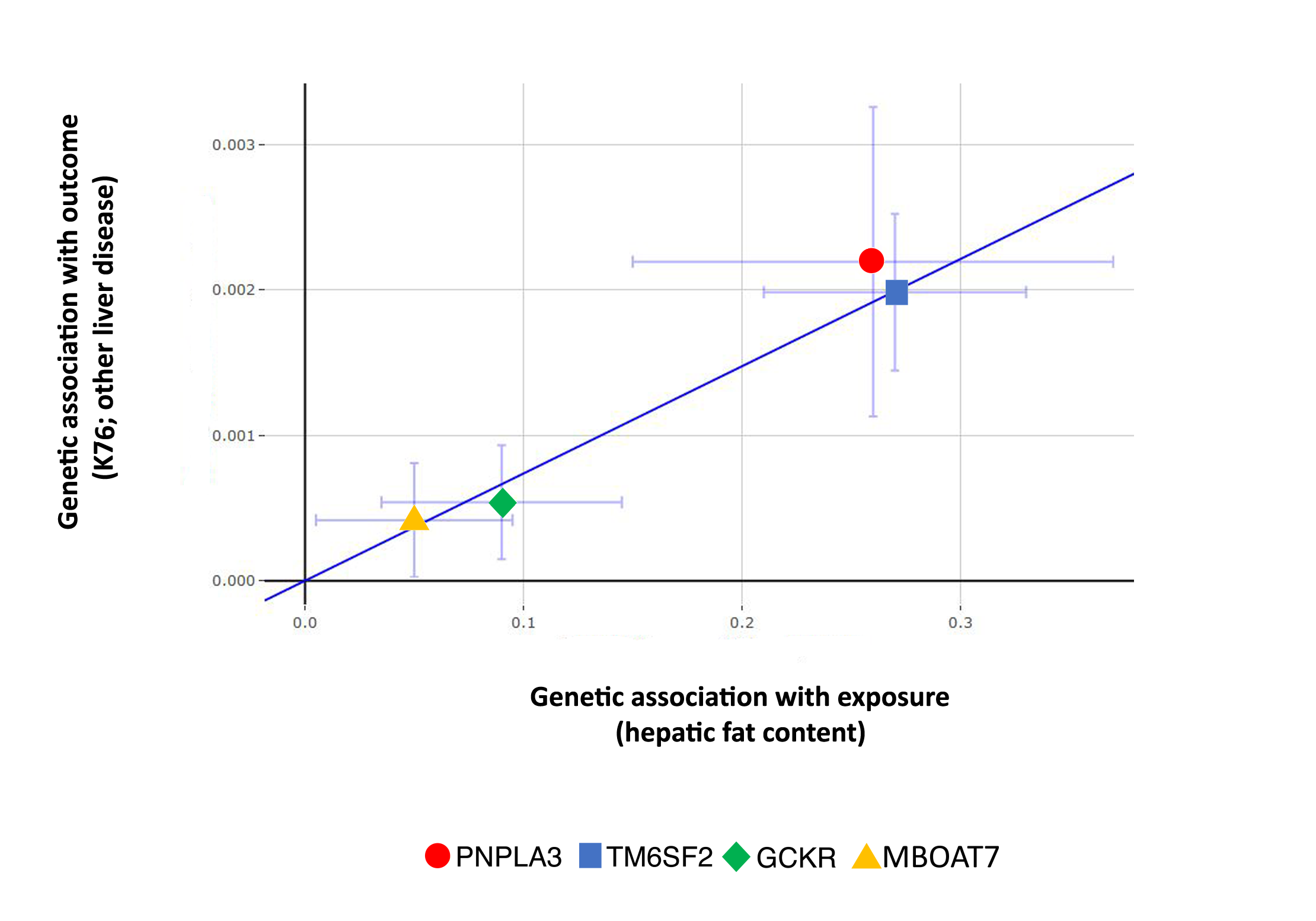

Supplement: Supplementary file 1 — Data S1. Supplementary methods. Data S2. Supplementary results. Table S1. Noninvasive fibrosis scores tested in the DHS for association with hepatic fat content. Table S2. Coefficient used to develop genetic risk scores (GRS) applied in the study cohorts (full models and sensitivity analysis conducted by removing one genetic variant at time). Table S3. Association of genetic variants included in the GRS (PNPLA3 rs738409 I148M, TM6SF2 rs58542926 E167K, GCKR rs1260326 P446L, and MBOAT7 rs641738) with liver damage and clinical parameters epidemiologically associated with NAFLD, in the LBC (N = 1515), SOS (N = 3329), and DHS (N = 4570). Estimates of β variant‐feature are reported for adjusted models. Table S4. Causal effect estimates of hepatic fat content, as evaluated by instrumental regression analysis by the 2SLS method, on metabolic and hepatic correlates of NAFLD in the LBC (n = 1515 at risk of NASH) and the DHS (n = 2736 from the general population). Table S5. Sensitivity analyses of IV regression analysis evaluating the causal role of hepatic fat on the hepatic and metabolic NAFLD features in the study cohorts with alternative GRS excluding one genetic risk variant per time. Estimate of causality ‐ β coefficient ‐ (95% confidence intervals) and P values are reported for adjusted models. Table S6. Associations of steatogenic variants with ICD‐code defined liver endpoints in 408 455 participants from UKBiobank. Table S7. Weights used in GRS construction in DHS. Table S8. Impact of the LYPLAL1 rs12137855 variant on hepatic and metabolic features of NAFLD in the DHS. Estimate of βvariant‐feature are reported for adjusted models. Table S9. Association of genetically determined hepatic fat with liver damage and metabolic features, using alternative GRS‐S1 and GRS‐S2. Table S10. Sensitivity analysis: causal effect estimates of hepatic fat content on hepatic and metabolic correlates of NAFLD in the LBC, stratified by modality of recruitment (Hepatology clinics vs. Baria [file JOIM-283-356-s001.docx]
